# A Rapid and Economical Method for Efficient DNA Extraction from Diverse Soils Suitable for Metagenomic Applications

**DOI:** 10.1371/journal.pone.0132441

**Published:** 2015-07-13

**Authors:** Selvaraju Gayathri Devi, Anwar Aliya Fathima, Sudhakar Radha, Rex Arunraj, Wayne R. Curtis, Mohandass Ramya

**Affiliations:** 1 Department of Genetic Engineering, SRM University, Kattankulathur, Tamilnadu, India; 2 Department of Chemical Engineering, Pennsylvania State University, University Park, Pennsylvania, United States of America; West Chester University of Pennsylvania, UNITED STATES

## Abstract

A rapid, cost effective method of metagenomic DNA extraction from soil is a useful tool for environmental microbiology. The present work describes an improved method of DNA extraction namely “powdered glass method” from diverse soils. The method involves the use of sterile glass powder for cell lysis followed by addition of 1% powdered activated charcoal (PAC) as purifying agent to remove humic substances. The method yielded substantial DNA (5.87 ± 0.04 μg/g of soil) with high purity (A_260/280_: 1.76 ± 0.05) and reduced humic substances (A_340_: 0.047 ± 0.03). The quality of the extracted DNA was compared against five different methods based on 16S rDNA PCR amplification, *Bam*HI digestion and validated using quantitative PCR. The digested DNA was used for a metagenomic library construction with the transformation efficiency of 4 X 10^6^ CFU mL^-1^. Besides providing rapid, efficient and economical extraction of metgenomic DNA from diverse soils, this method’s applicability is also demonstrated for cultivated organisms (Gram positive *B*. *subtilis* NRRL-B-201, Gram negative *E*. *coli* MTCC40, and a microalgae *C*. *sorokiniana* UTEX#1666).

## Introduction

Current estimates of microbial diversity reveal that 99% of the microorganisms present in nature are not cultivatable by standard techniques. The genetic information and biotechnological potential of the majority of organisms is therefore not revealed by conventional microbiological approaches [1]. Metagenomics, which involves direct cloning of environmental DNA, can obviate the need for cell cultivation, and thereby capture the genetic information from the total microbial community [2]. Since the abiotic and biotic factors of each habitat vary both spatially and temporally, a metagenomic DNA extraction method is needed that is broadly applicable, and yet standardized to permit relative comparisons.

The two important requirements for metagenomic DNA extraction are efficient cell lysis and purification of DNA from the complex milieu of an environmental sample. Cell lysis in soil samples has been accomplished by many methods that include chemicals such as sodium dodecyl sulphate (SDS) [3], chelex 100 [4], and guanidine thiocyanate [5], and physical methods such as bead beating [6], sonication [7], liquid nitrogen [8] and freeze thawing [9]. A traditional method dating back over 20 years uses a combination of freeze thawing and lysozyme [10]. However, it has been shown that these methods are often not sufficient to achieve complete cell lysis, or require the use of sophisticated equipment [11]. These methods are often time consuming and usually require an additional purification step before being subjected to molecular analysis. For soil samples in particular, purification to remove humic substances is necessary. Previously utilized purification agents include Polyvinylpolypyrrolidone (PVPP) [12], Sephadex G 200 [13], Q-Sepharose [14], electroelution [15] and silica gel [16]. Commercial kits are available for purification of metagenomic DNA; however, most are quite expensive.

Upon considering the limitations of previous methods (variable efficiency, time consuming and high cost), the current study focused on developing a rapid inexpensive method for extraction of metagenomic DNA with sufficient quantity and purity to be broadly suitable for metagenomic applications. Since, cell lysis and purification are the key steps in metagenomic DNA extraction; this study includes a particular focus on these two factors. Cell lysis is accomplished by homogenizing with glass powder that is obtained from laboratory waste glassware. Silica, the major component of ground glass powder, has been widely used for DNA extraction from various sources including soils and sediments [16], tissues and blood of transgenic animals [17] and plasmid from *E*.*coli* [18]. Autoclaved silica-based sand has been reported for extraction of fungal DNA [19], and glass powder along with skim milk was used for detection of *Phytophthora infestans* [20]. In a recent study by Radha et al. 2013 [21], a glass grinding step was included for direct colony PCR of various microalgae. However, a comparable glass powder based DNA extraction is not yet reported for metagenomic DNA from soils. Purification is accomplished with powdered activated charcoal (PAC), because it absorbs humic substances and allows the release of pure DNA [22, 11]. Hence, PAC was included in the extraction buffer of this study with the goal of eliminating the need for subsequent purification steps. An overall goal for the development of this method is to provide a procedure that is faster and less expensive than the method of *Yeates* et al 1998 [6] which utilize bead-beating, and multiple extractions for purification.

Diverse soils are tested along with cultivated examples for Gram positive bacteria, Gram negative bacteria and microalgae. The method is also compared with several alternative methods that utilize different homogenizing agent or equipment. We report the improved performance of this “powdered glass” method for 16S rDNA PCR amplification, quantitative PCR (qPCR) analysis, *Bam*HI restriction assay, and metagenomic library construction.

## Materials and Methods

### Chemicals, strains and plasmids

All chemicals of analytical or molecular biology grade were purchased from Sigma Aldrich, India. pUC19 plasmid and *E*.*coli* TOP 10 cells were purchased from Invitrogen Bio Services India Pvt. Ltd.

### Sample collection and analysis

Four different soil samples were collected for the study namely: (1) Garden soil from NageshwaraRao public park (13°2’11”N80°15’7”E) Chennai, India, (2) Sewage sludge from the Common effluent treatment plant (CETP) for tanneries, located at Pallavaram (12°57'44"N 80°8'8"E), Chennai, India, (3) Lake soil from the SRM University campus (12°49'25"N 80°2'39"E), Chennai, India, and (4) Compost sample from local house kitchen wastes. Samples were sieved using a 2 mm mesh and stored at -20°C for further analysis. No specific permissions were required to collect the samples from these locations and the field studies did not involve any endangered or protected species. Soil characteristics including texture, pH, electrical conductivity, organic matter, carbon, iron, cadmium and chromium were estimated as per APHA, (2005) [23] (S1 Table).

### Preparation of glass powder

A clean piece of broken borosilicate-based laboratory glass was ground finely using a mortar and pestle until it becomes a fine powder. The fine powder was autoclaved and stored in a sterile container, but may alternatively be pre-aliquoted and sterilized in amounts that correspond to a standardized procedure. The approximate size of the glass particles were measured by Field Emission-Scanning Electron Microscopy (Quanta FEI 200). The sample was examined on accelerating beam at a voltage of 10 kV at 10,000X magnification (S1 Fig).

### Metagenomic DNA extraction

Metagenomic DNA was extracted from all the four soil samples using six different methods. Method M1, M2, M3 and M4 were performed based on the previous studies (Table 1). Method 5 was performed using a commercially available kit (Fast DNA spin kit for soil; MP Biomedicals, Santa Ana, CA). M6 (powdered glass method) is the currently developed method, based largely on a prior method of extracting DNA from soil for subsequent PCR [6] as outlined in Table 2. In order to validate the superiority of the powdered glass method, the individual components of method M6 (glass powder and glass powder + powdered activated charcoal) were tested with method M2 (Glass beads). The other steps of method M2 were kept constant. The method M6 was also tested for genomic DNA extraction from Gram negative *E*. *coli* MTCC40, Gram positive *B*. *subtilis* NRRL-B-201 and eukaryotic microalgae *Chlorella sorokiniana* UTEX#1666 in order to confirm the suitability of the method for cultivatable organisms.

**Table 1:** Different methods used for extraction of metagenomic DNA from four different soils.

Method	Homogenizing agent	Homogenizing equipment	Processing time (h)	DNA Yield(μg/g of soil)
M1 [39]	Extraction buffer	Nil	5	1.29 ± 0.02
M2 [6]	Glass beads	Bead beater	7	3.42 ± 0.04
M3 [6]	Nil	Sonicator	7	1.47 ± 0.04
M4 [10]	Lysozyme	-70°C deep freezer and dryice ethanol bath	8	3.38 ± 0.05
M5 (Fast DNA SPIN kit)	As per the manufacturer’s protocol	Bead beater	1.5	3.51 ± 0.03
M6 (present study)	Glass Powder	Nil	1.5	5.87 ± 0.04

DNA yield represents the Average ± SD values of four different soils.

Nil represents there is no usage of homogenizing equipment.

**Table 2:** Metagenomic DNA extraction from soils using the currently developed powdered glass method M6 modified from [6].

Step	Procedure
1.	Take a clean, broken laboratory glass ware (borosilicate) and grind it using a pestle and mortar until it becomes a fine powder (Wear gloves and facemask). Transfer the finely ground powder to a container and sterilize by autoclaving at 121°C for 15 to 20 min or pre-aliquot and sterilize in amounts that correspond to a standardized procedure.
2.	Weigh 1 g of soil sample and 1 g of sterile glass powder, transfer in an autoclaved mortar and pestle and grind finely for about 5 min.
3.	Prepare enough DNA extraction buffer [100 mM Tris, 100 mM EDTA, 1.5 M NaCl (pH 8)]. Sterilize it by autoclaving at 121°C for 15 to 20 min or filter sterilization and store at room temperature. Weigh 10mg (1%) of powdered activated charcoal.
4.	Add 1 mL of DNA extraction buffer and 10mg of powdered activated charcoal to the soil- glass powder mixture and mix it by pipetting several times. Slowly transfer the contents into a 2 mL eppendorf tube.
5.	Incubate the tube at 65°C for 10 min in a water bath and then centrifuge at 12000g for 5 min at 4°C. Transfer 500 μL of the supernatant to a fresh 2 mL microfuge tube.
6.	Prepare enough quantity of 3M sodium acetate (pH 5.2) and 30% PEG (MW-8000). Sterilize by autoclaving at 121°C for 15 to 20 min or filter sterilization and store at room temperature.
7.	Add 100 μL of sodium acetate and 400 μL of PEG to the supernatant. Allow the mixture to precipitate at -20°C for 20 minutes in a deep freezer. Slowly thaw the tubes and then centrifuge at 12,000g, 5 min at 4°C.
8.	Discard the supernatant and re-suspend the pellet with 500 μL of autoclaved TE buffer (10 mM Tris, 1 mM EDTA pH 8).
9.	Prepare chloroform: isoamyl alcohol mixture in the ratio 24:1 and store it in a brown bottle at room temperature.
10.	Add equal volume (500 μL) of chloroform: isoamyl alcohol mixture and centrifuge at 12,000g for 5 min at 4°C.
11.	Transfer the aqueous phase to a fresh eppendorf and add 500 μL of ice cold isopropanol.
12.	Allow precipitation for 5 min at 4°C and centrifuge at 12,000g for 10 min at 4°C. Discard the supernatant and wash the precipitate with 70% ethanol. Centrifuge at 12,000g for 2 min at 4°C.
13.	Discard the supernatant, air dry the pellet and dissolve in 100 μL of TE buffer (pH 8).

### Assessment of yield and purity of the metagenomic DNA

**Fig 1:**
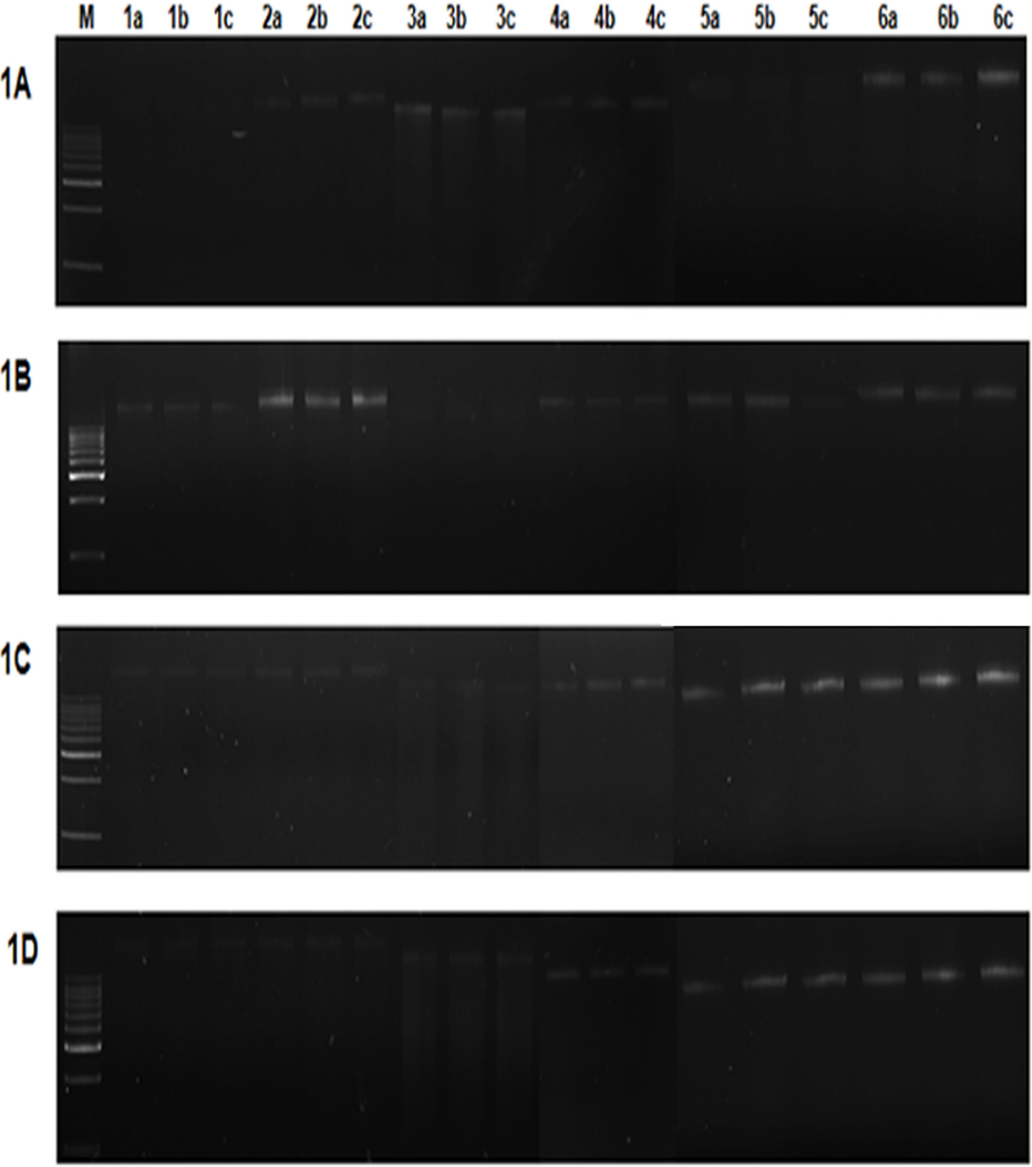
Gel electrophoresis of metagenomic DNA extracted by methods M1 to M6 for four different soils. Samples were electrophoresed on 0.8% agarose gel in 0.5X TBE buffer. **1A.** Garden soil; **1B**. Sewage sludge; **1C.** Lake soil; **1D.** Compost; Lane M represents 1Kb DNA ladder (Merck, India). Lanes 1 to 6 represents the methods M1 to M6 respectively. a,b,c represents the triplicates of corresponding method.

Equal volume (3 μL) of the extracted metagenomic DNA from all the samples for methods M1 to M6 were loaded in 0.8% agarose gel along with 3 μL of 1Kb DNA ladder (50 μg/mL) and the bands were visualized using UVP-Multidoc-It digital imaging system, CA, USA (Fig 1, S2 Fig). Purity and concentration of metagenomic DNA was determined by spectrophotometry analysis (UV-vis spectrophotometer, Eppendorf, NY). Concentration of the DNA (ng/μL) allowed calculation of Yield in μg per gram of soil = concentration of DNA (μg/μL)•volume used to suspend DNA (μL) /weight of soil (g) [24]. The absorbance A_260/280_ was used to determine protein contamination, and A_340_ as an indication of humic acid contamination.

### PCR amplification and quantitative PCR analysis

**Fig 2:**
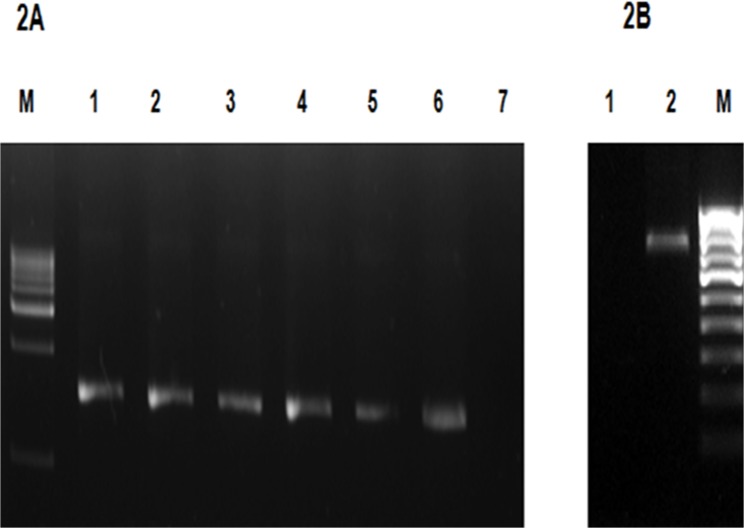
(A) Gel electrophoresis of PCR amplified 16S rDNA for the DNA extracted using the powdered glass method M6. Samples were analyzed on 1% agarose gel in 0.5X TBE buffer. Lane M: 1 Kb DNA ladder (Merck, India); Lane 1: Garden soil; Lane 2: Sewage sludge; Lane 3: Lake soil; Lane 4: Compost; Lane 5: *E*.*coli* MTCC 40; Lane 6: *Bacillus subtilis* NRRL-B-201. Lane 7: Negative control. **(B). Gel electrophoresis of PCR amplified *ITS*-2 for the DNA extracted using the powdered glass method M6.** Lane M: 100bp DNA ladder (Merck, India); Lane 1: Negative control; Lane 2: PCR amplification of *ITS* -2 region of *Chlorella sorokiniana* UTEX# 1666.

PCR amplification of the 16S rDNA was performed for all the methods (one sample per triplicate) using the universal forward primer B 27F (5′-AGAGTTTGATCCTGGCTCAG-3′) and the reverse primer U 1492R (5′-GGTTACCTTGTTACGACTT-3′). The reaction mix consisted of 50 ng of the metagenomic DNA as the template, 5 pmoles of each primer, 0.5 U *Taq* polymerase (GeNet Bio, Korea), 5 μL of 10X *Taq* buffer [10X buffer composition: Tris-HCl pH 9.0; PCR enhancers; (NH_4_)_2_SO_4_; 20 mM MgCl_2_] and 10 mM dNTP mix (NEB, USA). The final mixture was adjusted to 50 μL by addition of sterile, purified water. The amplification steps includes initial denaturation at 95°C for 5 minutes, 35 cycles of denaturation at 95°C for 1 min, annealing at 55°C for 30 s and extension at 72°C for 1.5 min with the final extension of 72°C for 7 min. 50 ng of genomic DNA from Gram positive and Gram negative bacteria was also amplified under similar conditions. 50 ng of the genomic DNA from the algae *Chlorella sorokiniana* UTEX#1666 was amplified using universal internal transcribed spacer 2 (*ITS*-2) primers: forward (5′-AGGAGAAGTCGTAACAAGGT-3′) and reverse (5′-TCCTCCGCTTATTGATATGC-3′). The conditions used for PCR were initial denaturation at 95°C for 5 min, 35 cycles of denaturation at 95°C for 1 min, annealing at 55°C for 30 s, extension at 72°C for 1 min and a final extension of 72°C for 5 min. Amplification products were confirmed by loading 5 μL of samples along with 1Kb DNA ladder on 1% agarose gel (Fig 2, S3 Fig). PCR efficiency of the DNA isolated using the powdered glass method M6 was analyzed for a single soil sample (sewage sludge) by qPCR (Lightcycler 480 system, Roche Life Sciences, US) using the primers for 16S rDNA: forward (5′-AAGCAACGCGAAGAACCTTA-3′) and reverse (5′-ACCACCTGTCACCTCTGTCC-3′). The reaction volume (10 μL) consisted of DNA (10 ng to 100 pg), primers (5 pmoles) and 5 μL of 2x SYBR Green I master mix and the reaction was performed in triplicate. The reaction without the template served as a non-template control (NTC). The amplification conditions were 95°C for 7 min as initial denaturation followed by 40 cycles of 95°C for 20 s, 55°C for 20 s and 72°C for 20 s (S6 Fig).

### Partial restriction digestion and metagenomic library construction

**Fig 3:**
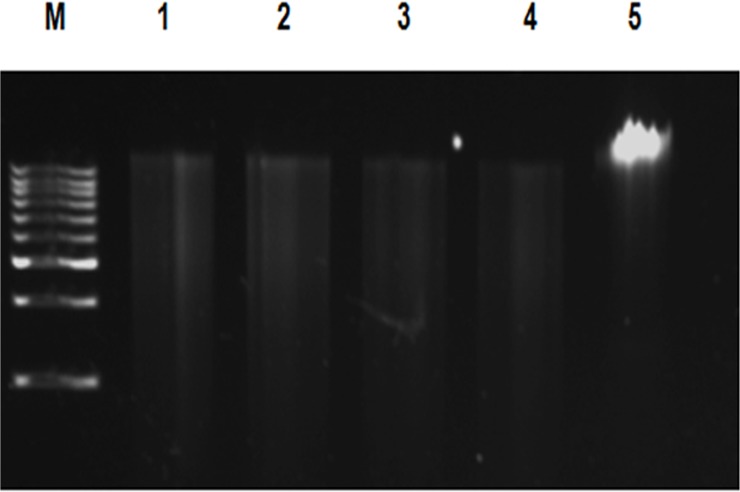
Gel electrophoresis of partial restriction digestion of the metagenomic DNA extracted by powdered glass method (M6) using *Bam*HI. Samples were analyzed on 0.8% agarose gel in 0.5X TBE buffer. Lane M: 1Kb DNA ladder (Merck, India); Lane 1: Garden soil; Lane 2: Sewage sludge; Lane 3: Lake soil; Lane 4: Compost. Lane 5: Undigested DNA.

Partial restriction digestion was performed using the enzyme *Bam*HI (Fermentas, Germany) for DNA extracted by all the methods (one sample per triplicate) except M3 to examine the suitability of the methods for downstream DNA manipulation. 1 μg of the DNA template was digested with 1 U of the enzyme in a 50 μL reaction containing 5 μL of 10X assay buffer [1X buffer composition: 10 mM Tris-HCl pH 8.0; 5 mM MgCl_2_; 100 mM KCl; 0.02% TritonX-100; 0.1 mg/mL BSA] for 20 min at 37°C followed by heat inactivation of the enzyme at 70°C for 10 min. 5 μL of the digested products were analyzed on 0.8% agarose gel along with 1Kb DNA ladder (Fig 3, S4 Fig). A soil metagenomic library was constructed for the DNA isolated by powdered glass method (M6) using the pUC19 vector in the following manner: 2 to 10 kb fragments from the partially digested DNA were gel purified using EZ-Spin PCR purification column (Biobasic Inc., Canada). pUC19 plasmid was linearized with *Bam*HI and dephosphorylated using 10 U of calf intestinal alkaline phosphatase. Linearized vector was ligated with the insert [vector: insert Molar ratio (1:3)] using 200 U of T4 DNA ligase (NEB, USA) in a 10 μL reaction containing 1 μL of ligase buffer [1X buffer composition: 50 mM Tris-HCl, pH 7.5; 10 mM MgCl_2_; 1 mM ATP; 10 mM DTT] at 16°C overnight. 5 μL of the ligation mixture was then added to 100 μL of chemically competent *E*.*coli* TOP 10 cells and incubated on ice for 20 min. Heat shock was applied at 42°C for 90 s, then transferred to ice for 5 min followed by addition of 1 mL of Luria Bertani (LB) broth and cells were allowed to grow at 37°C for 1 h. 100 μL of cells were plated in LB media containing ampicillin (50 μg/mL), IPTG (0.1 mM) and X-gal (40 μg/mL). The recombinant colonies were re-plated on LB plate with ampicillin (50 μg/mL).

### Statistical analysis

The experiments were performed in triplicate and the mean and standard deviations were estimated for each experiment. Analysis of variance (One-way ANOVA) was performed with the software GraphPad Prism 5.0 to determine the significant effects of extraction methods and soil types on DNA yield. Paired T-test analysis was performed for the pairwise comparison of DNA yields obtained by other methods (M1 to M5) against the powdered glass method M6 for different soil samples. The comparisons were considered significant if p<0.05.

## Results

### Extraction of metagenomic DNA from different samples

Metagenomic DNA was extracted from different soil samples using earlier methods (M1, M2, M3, M4) and commercially available Fast DNA spin kit for soil (MP Biomedicals, CA) (M5) and compared with the currently developed powdered glass method (M6). Upon analysis of the extracted soil samples on 0.8% agarose gels, method M6 is shown to produce intact and bright bands for all samples (Fig 1A, 1B, 1C and 1D). Method M5 showed bright bands for DNA extracted by lake and compost soils (Fig 1C and 1D), whereas M4 produced bright bands for garden and compost soils (Fig 1A and 1D). Further illustrating the variation of results with different soils, M2 showed bright bands for garden soil and sewage sludge (Fig 1A and 1B). Methods M1 and M3 did not produce bright bands for any of the samples.

### Comparison of DNA yield and purity using various methods

The amount of DNA (μg/g) for the four different soils is depicted in Table 3 and S3 Table. Our improved powdered glass method M6 produced high average yield of DNA/g of soil (5.87 ± 0.04 μg) followed by methods M5, M2 and M4 (3.51 ± 0.03, 3.42 ± 0.04, 3.38 ± 0.05 μg/g respectively). Methods M3 and M1 produced the lowest DNA yield (1.47 ± 0.04, 1.29 ± 0.02 μg/g respectively) per gram of soil. Analysis of variance (One-way ANOVA) revealed that the different DNA extraction methods as well as various soil types had significant effects on the DNA yield (P<0.0001). Statistically significant differences in DNA yields were obtained based on T-test analysis for pairwise comparison of each method against the powdered glass method M6 (Table 3)).

**Table 3:** Assessment of yield of metagenomic DNA obtained by methods M1 to M6 for different soils.

Yield (μg/g of soil)				
Methods	Garden soil	Sewage sludge	Lake soil	Compost
**M1**	1.34 ± 0.01****	1.49 ± 0.02****	1.42 ± 0.02***	0.93 ± 0.03****
**M2**	4.92 ± 0.03**	5.01 ± 0.04***	1.52 ± 0.04***	2.24 ± 0.07****
**M3**	3.63 ± 0.06***	0.56 ± 0.05****	1.08 ± 0.02***	0.62 ± 0.03****
**M4**	5.06 ± 0.05*	2.06 ± 0.06****	2.25 ± 0.04****	4.15 ± 0.06***
**M5**	2.01 ± 0.03***	2.56 ± 0.05****	4.58 ± 0.02***	4.89 ± 0.03 ^ns^
**M6**	5.48 ± 0.03	7.81 ± 0.04	5.19 ± 0.06	4.98 ± 0.04

DNA concentration (μg/mL) was quantified in a spectrophotometer (A_260_ nm).

Yield in μg per gram of soil = concentration of DNA (μg/μL). Volume used to suspend DNA (μL) /weight of soil (g)

P values were generated by Graph Pad Prism 5.0 software using paired T-test analysis.

P value < 0.05 is considered to be statistically significant. DNA extraction methods performing worse at a statistically significant level as compared with the best performing method M6 are indicated by asterisks

**** indicates P < 0.0001

*** indicates P < 0.001

** indicates P < 0.01

* indicates P < 0.05

^ns^ indicates P > 0.05 which is statistically non-significant.

An assessment of the extracted DNA purity demonstrates that the method M6 provides more pure DNA with an average absorbance ratio (A_260/280_) of 1.76 ± 0.05, as compared to absorbance ratios lower than 1.5 for the other methods. The A_340_ absorbance value was taken as an indication of humic acid contamination rather than A_230_ because it has less overlap with the absorbance of DNA which is measured at A_260_ and the associated use of A_260_/A_230_ as a typical ratio indicator of DNA purity [22]. The average A_340_ value for method M6 is low (0.047 ± 0.03) as compared to the other methods (Table 4, S4 and S5 Tables) which is consistent with the recent report of Sharma et al. (2014) [22] that demonstrated effective use of PAC for the reduction in humic substances.

**Table 4:** Assessment of purity of metagenomic DNA by methods M1 to M6 for different soils.

A_260/280_					A_340_			
Methods	Garden soil	Sewage sludge	Lake soil	Compost	Garden soil	Sewage sludge	Lake soil	Compost
**M1**	1.14 ± 0.02	1.22 ± 0.03	1.22 ± 0.09	1.29 ± 0.04	0.09 ± 0.03	0.08 ± 0.02	0.09 ± 0.03	0.08 ± 0.01
**M2**	1.53 ± 0.06	1.48 ± 0.03	1.56 ± 0.01	1.40 ± 0.07	0.06 ± 0.02	0.07 ± 0.03	0.08 ± 0.02	0.06 ± 0.04
**M3**	1.32 ± 0.03	1.25 ± 0.05	1.34 ± 0.01	1.37 ± 0.06	0.07 ± 0.01	0.08 ± 0.03	0.06 ± 0.04	0.06 ± 0.05
**M4**	1.33 ± 0.05	1.34 ± 0.06	1.46 ± 0.09	1.48 ± 0.03	0.09 ± 0.04	0.08 ± 0.02	0.08 ± 0.03	0.09 ± 0.02
**M5**	1.60 ± 0.04	1.62 ± 0.08	1.56 ± 0.03	1.50 ± 0.07	0.06 ± 0.04	0.06 ± 0.03	0.07 ± 0.08	0.08 ± 0.01
**M6**	1.82 ± 0.08	1.72 ± 0.04	1.78 ± 0.02	1.73 ± 0.05	0.04 ± 0.03	0.06 ± 0.04	0.05 ± 0.01	0.04 ± 0.06

A_260/280_ represents protein contamination. A_340_ is an indication for humic acid presence.

An assessment of the efficiency of DNA extraction from soil for glass powder relative to glass beads, and the impact of combining the PAC as a single step is presented in Table 5, S5 Fig and S6 Table. The average yield of DNA using glass beads was 2.1 ± 0.08 μg/g of soil; much lower than glass powder (5 ± 0.11 μg/g of soil). The addition of PAC to the glass powder did not adversely affect the DNA yield with an average yield of 5.4 ± 0.04 μg/g for the four soil types analyzed. In addition, the purity obtained by the use of glass powder combined with PAC was higher when compared to the use of glass beads or glass powder alone.

**Table 5:** Assessment of yield and purity of the DNA extracted by method M2 and its modification (glass beads, glass powder and glass powder + powdered activated charcoal).

Method/Soil	Glass Beads			Glass Powder			Glass Powder + PAC		
	DNA yield (μg/g)	A_260/280_	A_340_	DNA yield (μg/g)	A_260/280_	A_340_	DNA yield (μg/g)	A_260/280_	A_340_
**Garden soil**	3.3 ± 0.05 ***	1.50 ± 0.03	0.06 ± 0.04	5.5 ± 0.07 ***	1.50 ± 0.01	0.03 ± 0.02	6.2 ± 0.04	1.80 ± 0.03	0.05 ± 0.02
**Sewage sludge**	1.2 ± 0.03 ****	1.45 ± 0.04	0.06 ± 0.03	5.8 ± 0.07 ^ns^	1.52 ± 0.03	0.06 ± 0.05	5.9 ± 0.03	1.69 ± 0.04	0.03 ± 0.02
**Lake soil**	1.0 ± 0.07***	1.30 ± 0.02	0.13 ± 0.07	4.2 ± 0.15 ^ns^	1.42 ± 0.04	0.07 ± 0.04	4.4 ± 0.03	1.73 ± 0.04	0.03 ± 0.02
**Compost**	3.0 ± 0.18 ***	1.44 ± 0.05	0.09 ± 0.06	4.1 ± 0.15 **	1.50 ± 0.04	0.08 ± 0.04	5.0 ± 0.09	1.74 ± 0.06	0.04 ± 0.05

DNA concentration (μg/mL) was quantified in a spectrophotometer (A_260_ nm).

Yield in μg per gram of soil = concentration of DNA (μg/μL). Volume used to suspend DNA (μL) /weight of soil (g).

A_260/280_ represents protein contamination. A_340_ is an indication for humic acid presence.

PAC represents powdered activated charcoal.

P values were generated by Graph Pad Prism 5.0 software using paired T-test analysis.

P value < 0.05 is considered to be statistically significant. DNA extraction methods performing worse at a statistically significant level as compared with the best performing method (Glass powder + PAC) are indicated by asterisks

**** indicates P < 0.0001

*** indicates P < 0.001

** indicates P < 0.01

^ns^ indicates P > 0.05 which is statistically non-significant.

Further, as an indicator of the superiority of our improved methodology, method M6 also yielded a higher concentration and higher purity DNA when tested for its applicability on cultivatable organisms. The concentration (ng of DNA/μL) and purity A_260/280_ were respectively found to be 71.1 ± 0.25 ng/μL and 1.80 ± 0.04 for Gram negative *E*. *coli* MTCC40, 42.2 ± 0.42 ng/μL and 1.73 ± 0.02 for Gram positive *B*. *subtilis* NRRL-B-201 and 39.0 ± 0.68 ng/μL and 1.62 ± 0.07 for *C*. *sorokiniana* UTEX# 1666 (S2 Fig). The presence of peptidoglycan in the cell wall of Gram positive bacteria and glycoprotein rich cell wall of microalgae likely contributes to the lower yield of DNA as compared to Gram negative bacteria.

### PCR Amplification and quantitative PCR analysis of ribosomal RNA genes

DNA samples were tested for their suitability for PCR amplification of 16S rDNA. Powdered glass method (M6) produced an amplification product of 1.5 kb for all the samples including Gram positive and Gram negative bacteria (Fig 2A). Genomic DNA of *C*. *sorokiniana* UTEX# 1666 obtained by method M6 was also amplified using algal specific *ITS*-2 primers producing a PCR product of 800 bp (Fig 2B). Methods M1, M3 and M4 did not provide amplification for any of the soil samples indicating that those methods would require further purification of DNA to remove humic substances. M2 and M5 provided amplification for a few samples (S3 Fig); however, these methods would require additional purification to be suitable for all the soil types. The qPCR efficiency (indicative of the exponential increase in DNA during successive PCR cycles) for the DNA isolated using powdered glass method (M6) was 1.986 (99.3%) with a slope of -3.356 (S6 Fig).

### Partial restriction digestion and metagenomic library construction

All the soil DNA samples extracted by powdered glass method (M6) were partially digested with the enzyme *Bam*HI for metagenomic library construction (Fig 3). Method M5 was also suitable for digestion of all DNA extracts, where the other methods failed to achieve digestion for at least one of the samples (S4 Fig). Method M3 was not subjected to restriction digestion because of the shear-degraded, sonicated DNA. The suitability of the other methods for restriction digestion and 16S rDNA PCR is presented in S2 Table. The partially digested DNA by powdered glass method M6 was subjected to metagenomic library construction using pUC19 vector and colonies were obtained with the transformation efficiency of 4 X 10^6^ CFU mL^-1^. This confirms the efficacy of the method for the required downstream metagenomic DNA processing including PCR, restriction digestion and library construction.

## Discussion

Various methods are available for metagenomic DNA extraction based on chemical or mechanical lysis of microbial cells present in the soil. Among these methods, glass bead beating is considered to be an effective technique for metagenomic DNA extraction [6]. This method has also been modified in previous reports to be suitable for different soil types [25]. Commercial kits such as Fast DNA SPIN kit for soil, MP Biomedicals, Santa Ana, CA) and Ultra Clean Mo Bio Soil DNA isolation kit are also based on the method of bead beating. Even though this method was adopted for different soils, it requires ‘bead beater’ for cell lysis and the method’s efficiency depends on the size of beads and duration of agitation. While ‘bead beating’ and our improved method M6 both utilize glass, the mechanism of cell breakup is fundamentally different. Bead mills and other suspension-based cell breakup approaches rely on the fluid-dynamic shear that results in flows between particles [26], or more accurately, the mechanisms of dissipating the imparted energy at the turbulent length scales of the cells [27]. In contrast, the improved method M6 utilizes direct mechanical grinding of the soil and glass shards (Table 2). Therefore, in contrast to shear forces of bead beating, the mortar and pestle grinding represents a direct physical maceration, which is apparently more effective means of disrupting the cells in a metagenomic soil sample based on DNA recovery yield (Table 3). A potential reason for reduced performance of cell disruption in soil samples by bead beating could result from increased viscosity due to the presence of a high concentration of insoluble materials during the beating process [28].

Our improved method M6 involves the fine grinding of soil samples with the glass powder which takes less than 5 min. In addition to providing an excellent mechanical force for cell breakup, we suggest that enhanced DNA adsorption on the finely powdered silica may also be playing a role [18]. Upon grinding, the cells are lysed and the DNA is adsorbed by the silica particles which are subsequently extracted by addition of extraction buffer [21, 16]. The size of glass powder varies from 223 to 261 nm. This extremely small size of powdered glass relative to typical glass milling beads (~100 microns) provides greatly enhanced surface area as well as freshly fractured silica surfaces to contribute to DNA adsorption. The adsorption of DNA to silica has been intensely studied in relevance to sequencing analysis and microfluidics [29], and reports the advantage of large silica surface area for DNA extraction [30]. This behavior of DNA adsorption to glass has the potential to effect the application of these methods for PCR amplification and sequencing of the environmental samples to study microbial diversity and functionality in complex communities [16]. Li et al. 2015 [31] reported that the use of silica columns improves viral sequence recovery when compared to other methods of DNA extraction. Silica nanoparticles have been fabricated for the effective immobilization and sensitive sequence-specific detection of DNA [32]. An observation to be validated using the above methodology is its efficiency in the recovery of high molecular weight DNA (>20Kb) for use in large sized libraries. For this behavior to not affect metagenomic analysis, one must assume that the process of cell disruption and subsequent shearing of DNA is similar for different microorganisms. We suggest that the mechanical grinding of the method (M6), as compared to hydrodynamic shearing described above, may provide for a more representative sampling of the microbial populations. Future work combining defined mixtures of microorganisms to sterile soil could address this issue.

The efficiency of using powdered glass in mechanical cell lysis and enhanced surface area is confirmed by the yield of DNA which is much higher in method M6 than the other methods. Another advantage of this methodology is the inclusion of PAC in the extraction buffer to minimize additional purification steps. PAC is very porous with a vast surface area which allows the absorption of humic substances, lignin sulphonate, tannic acid, heavy metals and non-degradable coloured substances [33]. PAC has previously been used as a purifying agent in other reports of soil DNA extraction [11, 22]. In the present study, 1% PAC was added directly to the extraction buffer in order to absorb contaminating components of the metagenomic DNA that would otherwise inhibit subsequent DNA manipulation. This simplifies the need for subsequent column purification. Polyethylene glycol (30% PEG) has been used in other metagenomic DNA extraction methods because it does not co-precipitate humic acids with the DNA as occurs with ethanol or isopropanol precipitation [34]. In the current procedure (M6), the humic substances are precipitated in a simple intermediate step using chloroform and isopropanol as reported previously [35].

The powdered glass method (M6) is compared with four other previously reported methods and a commercial kit. All the methods follow the same basic steps: cell lysis and precipitation of DNA but they differ from each other on the homogenization approach and duration of the total protocol (Table 1). Methods M2, M3, M4 and M5 require homogenizing equipment for cell lysis whereas M1 is based on chemical lysis and M6 is based on glass powder grinding. Among all the methods studied, powdered glass method (M6) shows higher efficiency of cell lysis during DNA extraction which is evidenced in terms of DNA yield. The methods were also tested for four different soil samples that that differed in texture, pH, percentage of organic matter and the amount of metals (S1 Table). The presence or absence of clay and other particulate matter present in each soil is an important consideration because they tend to decrease the DNA yield by adsorbing the free DNA [36, 37]. Grinding with high surface area powdered glass is expected to out-compete such adsorption [38]. We feel that this characteristic helps contribute to the robust performance of method M6 for different soil types.

As noted above, methods M1 to M4 did not achieve either high yield or purity. Though method M1 [39] did not require any sophisticated equipment for cell lysis, it failed to meet high purity and yield for some of the soil samples, while method M3 apparently suffered from excessive shearing of the DNA due to sonication. Method M2 based on bead beating is often recommended as the most effective method; however, in our study, this method did not yield pure DNA for all the soil samples. Method M2 also requires the bead beater equipment for the cell lysis. Method M4 that used a combination of lysozyme and freeze thaw did not reduce the humic acid content. The Fast DNA kit for soil resulted in a better DNA yield and purity but still contained humic acid residues. Other reports also demonstrate the failure of commercial resins to provide DNA free from humic acids as they compete with the DNA for column binding [40, 41].

Downstream applications such as PCR amplification and restriction digestion involve enzymatic reactions that can be significantly inhibited by humic acids. Humic substances chelate the Mg^2+^ ions required for the activity of *Taq* polymerase, restriction enzymes and ligases [42]. The DNA isolated using methods M1 to M4 did not provide sufficiently pure DNA for further studies. Kit based method M5 provided DNA suitable for the restriction digestion assay; however, it was not found suitable for PCR amplification when tested on different soil samples. The results reveal that the methods M1 to M5 requires further purification of the DNA for molecular analysis. The DNA isolated using the powdered glass method M6 displays a high qPCR efficiency; greater than 99%, where the accepted PCR efficiency for qPCR analysis ranges from 90 to 110 percent (Roche Life Sciences, US). The suitability of the DNA for PCR, qPCR, restriction digestion assay and metagenomic library construction confirms that sufficient purity was achieved using method M6; a simplified method that integrates cell lysis and DNA purification steps (Table 2).

Despite the diversity in the sources of the soil samples, our improved powdered glass method (M6) produced high yield of DNA, demonstrating broader applicability of the method. The average DNA yield of the four soil samples was 5.87 μg/g which was considerably higher than the other methods (Table 1). In addition, protein and humic acid contamination was low when compared to the other tested methods. The purity of the metagenomic DNA obtained by this method M6 was further substantiated by facile construction of a metagenomic library. Further, this method was also successfully used for DNA extraction from Gram positive, Gram negative bacteria and also microalgae. The statistically significant improved efficiency of DNA extraction invariably contributes to downstream manipulations by effectively reducing the contaminants.

As a final added advantage, the overall processing time for powdered glass method (M6) was shorter than the other tested methods. The earlier reported methods (M1 to M4) took about 5 to 7 h for processing while the method M6 requires only 1.5 h which was considerably shorter than the recently reported method of Sagar et al. (2014) [35]. The commercial kit (method M5) took the least time for DNA extraction (Table 1); however, the cost of processing 1 g of soil for a single reaction is about $8 (US) which is quite high if large numbers of samples are to be processed. The use of glass powder is an economical method for cell lysis that does not require sophisticated equipment or enzymes for cell lysis.

## Conclusion

A robust method of DNA extraction from soil for metagenomic analysis and cultivated organisms is demonstrated which uses glass powder for cell lysis and powdered activated carbon for purification. This method is rapid, efficient and economical, and found to be robustly applicable for DNA extraction where intact and pure DNA is required, and may include additional advantages for providing a representative sampling of the metabiome population.

## Supporting Information

S1 Fig Size determination of the glass powder by FE-SEM analysis. (DOC)

S2 Fig Gel electrophoresis of genomic DNA isolated by method M6 for Gram positive, Gram negative bacteria and microalgae. (DOC)

S3 Fig Gel electrophoresis of PCR amplified 16S rDNA for the metagenomic DNA extracted by methods M1 to M5. (DOC)

S4 Fig Gel electrophoresis of partial restriction digestion of the metagenomic DNA extracted by methods M1, M2, M4 and M5 using *Bam*HI. (DOC)

S5 Fig Gel electrophoresis of metagenomic DNA extraction by the glass beads, glass powder and glass powder + powdered activated charcoal for four different soils. (DOC)

S6 Fig qPCR analysis for method M6. (DOC)

S1 Table Characteristics of the four different soil samples. (DOC)

S2 Table Suitability of the DNA samples extracted by methods M1 to M6 for 16S rDNA PCR amplification and partial restriction digestion by *Bam*HI. (DOC)

S3 Table Triplicate values for DNA DNA yield (μg/g of soil) for methods M1 to M6. (DOC)

S4 Table Triplicate values for A_260/280_. (DOC)

S5 Table Triplicate values for A_340_. (DOC)

S6 Table Triplicate values for DNA yield (μg/g of soil) for method M2 and its modification. (DOC)
