# Supplementary material for: A Rapid and Economical Method for Efficient DNA Extraction from Diverse Soils Suitable for Metagenomic Applications
Source: PLoS One. 2015 Jul 13;10(7):e0132441. doi: 10.1371/journal.pone.0132441 (PMC4500551; doi:10.1371/journal.pone.0132441)
Supplement: S1 Fig — (DOC) [file pone.0132441.s001.doc]

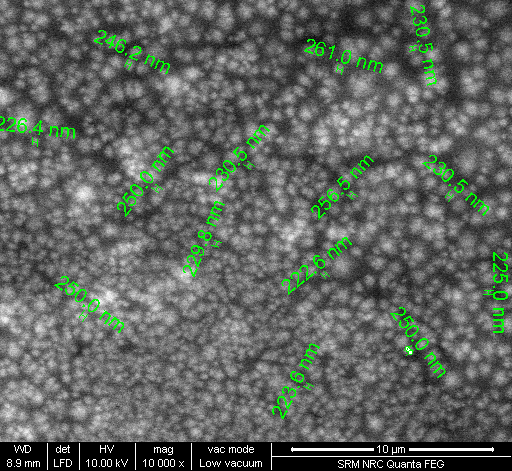


**S1 Fig.** The approximate size of the glass particles as measured by Field Emission-Scanning Electron Microscopy (FE-SEM) [Quanta FEI 200]. The sample was examined on accelerating beam at a voltage of 10kV at 10,000X magnification.
