# Supplementary material for: A Rapid and Economical Method for Efficient DNA Extraction from Diverse Soils Suitable for Metagenomic Applications
Source: PLoS One. 2015 Jul 13;10(7):e0132441. doi: 10.1371/journal.pone.0132441 (PMC4500551; doi:10.1371/journal.pone.0132441)
Supplement: S2 Fig — (DOC) [file pone.0132441.s002.doc]

**S2 Fig. Gel electrophoresis of genomic DNA isolated by method M6 for Gram positive, Gram negative bacteria and microalgae.** Samples were analyzed in 0.8% agarose gel in 0.5X TBE buffer. Lane M: 1Kb DNA ladder (Merck, India); Lane 1: *E.coli* MTCC40; Lane 2: *B. subtilis* NRRL-B-201; Lane 3: *C. sorokiniana* UTEX#1666


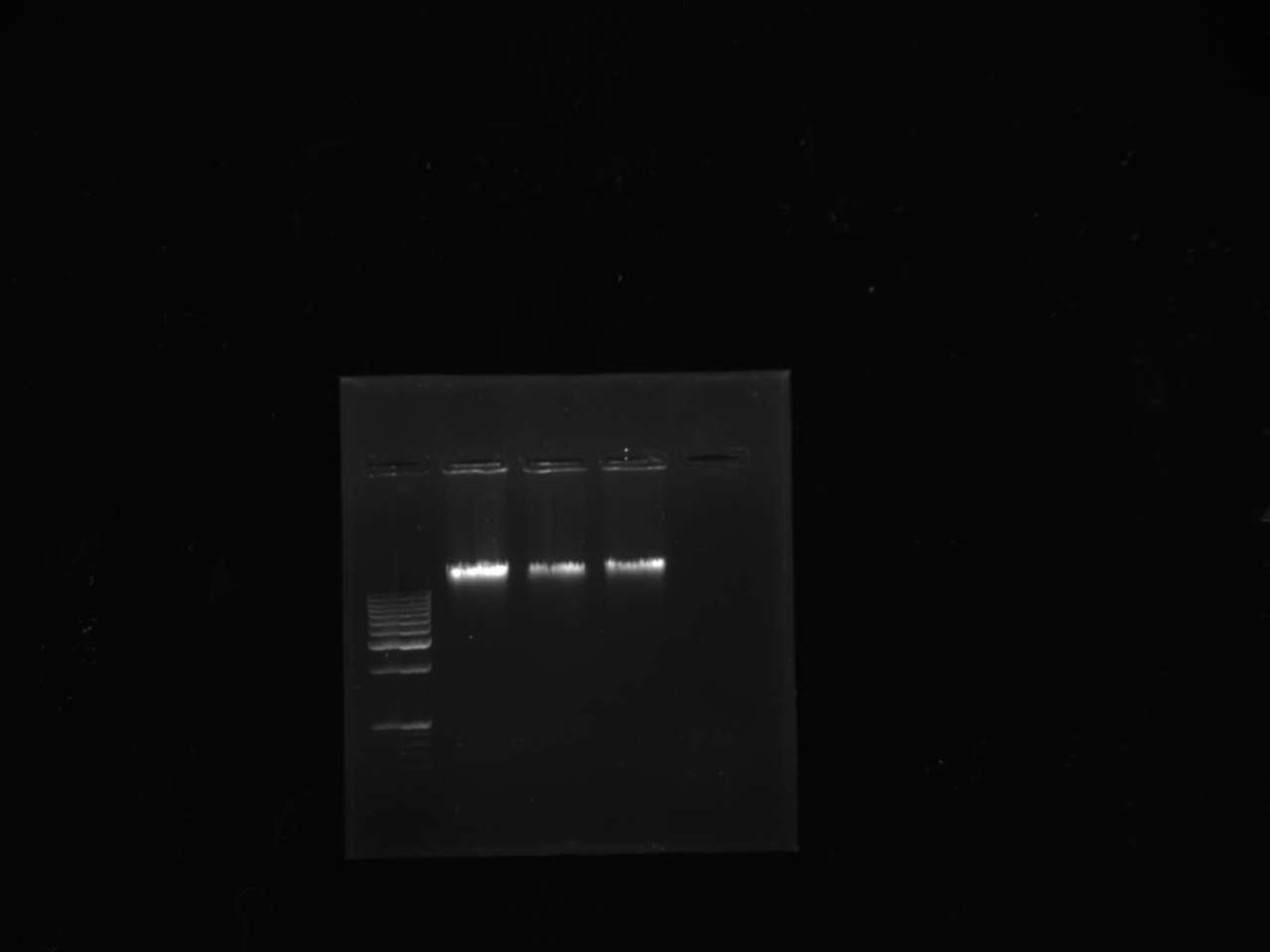


**M 1 2 3**
