# Supplementary material for: A Rapid and Economical Method for Efficient DNA Extraction from Diverse Soils Suitable for Metagenomic Applications
Source: PLoS One. 2015 Jul 13;10(7):e0132441. doi: 10.1371/journal.pone.0132441 (PMC4500551; doi:10.1371/journal.pone.0132441)
Supplement: S3 Fig — (DOC) [file pone.0132441.s003.doc]

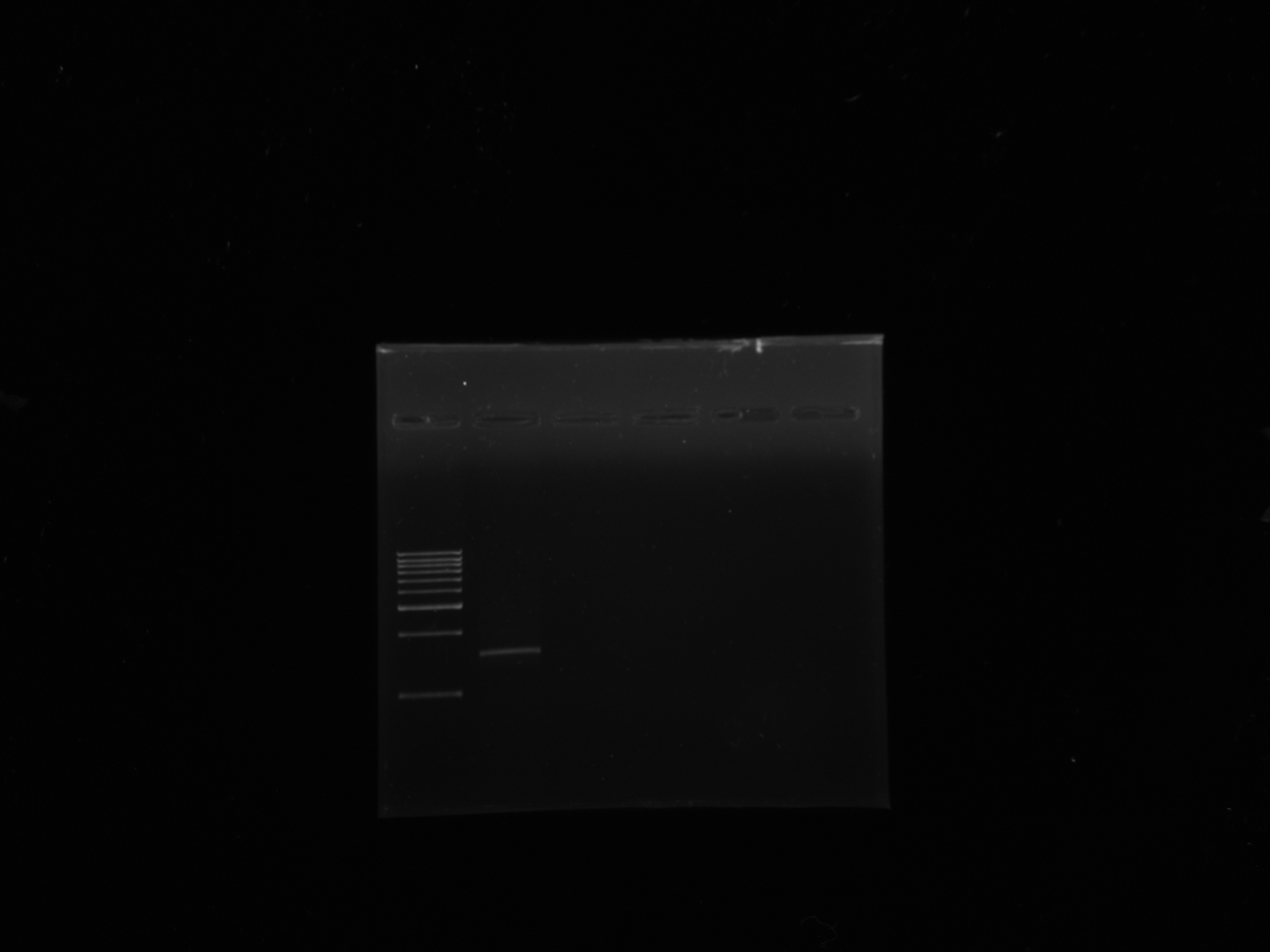

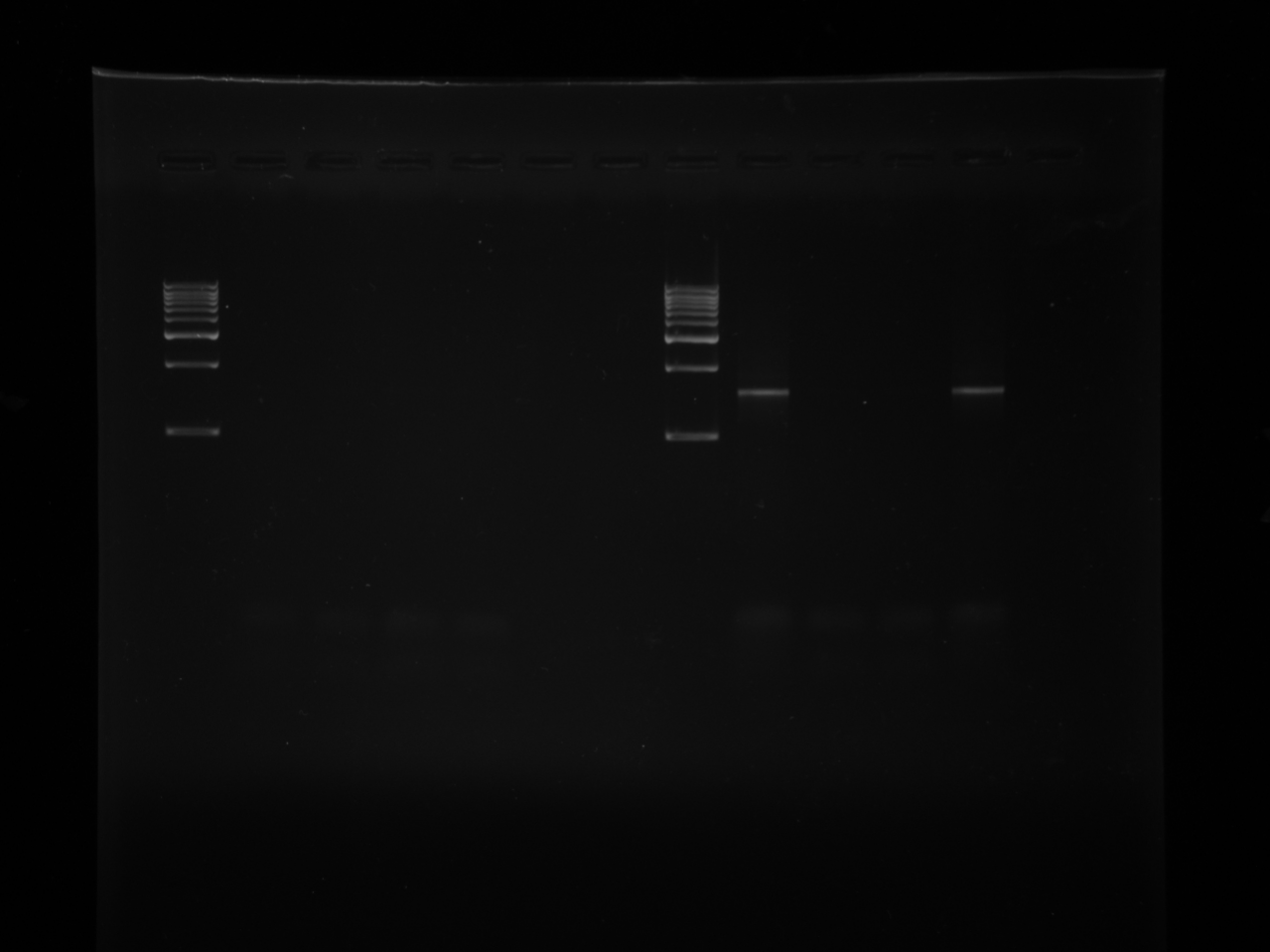

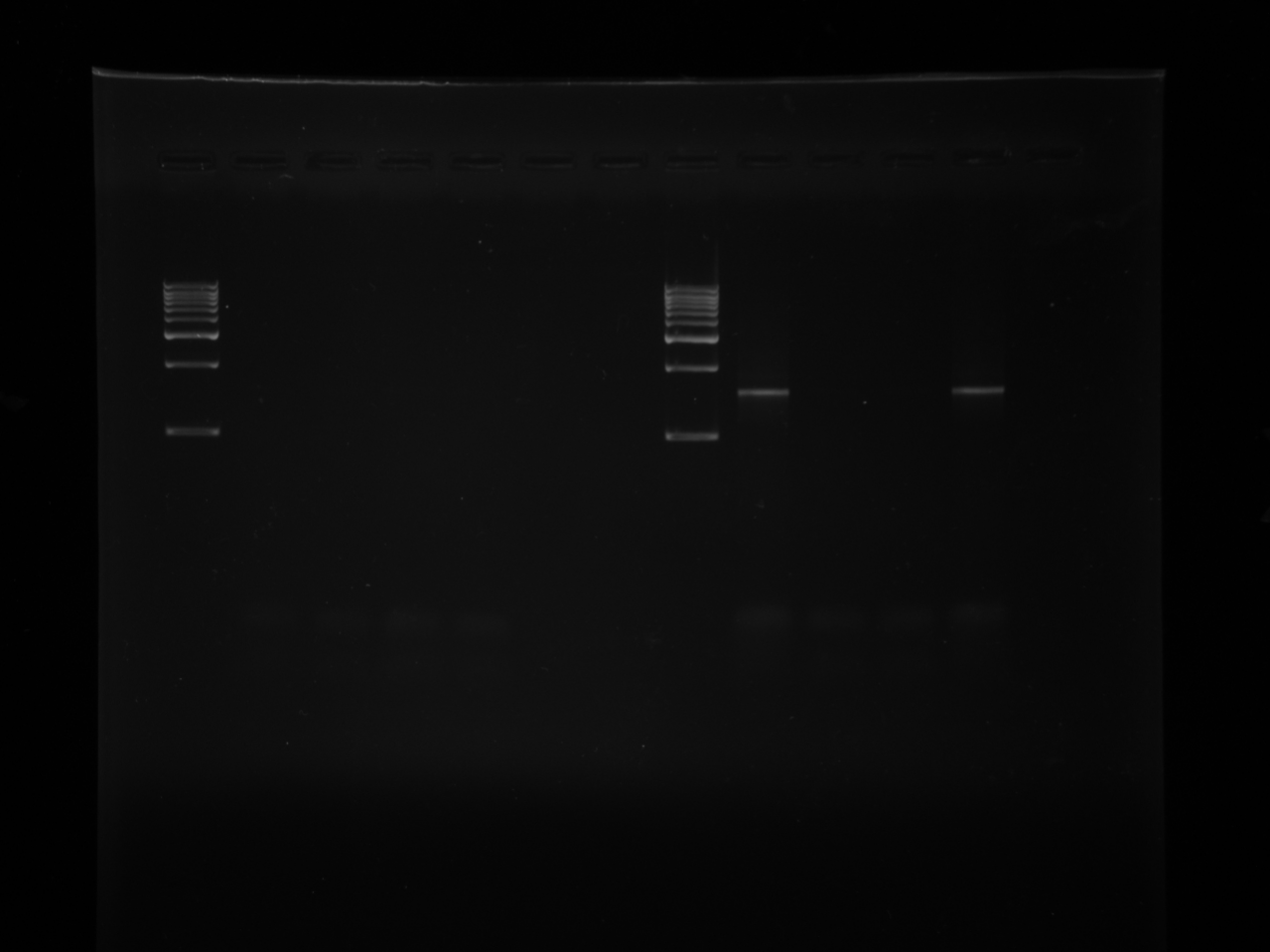

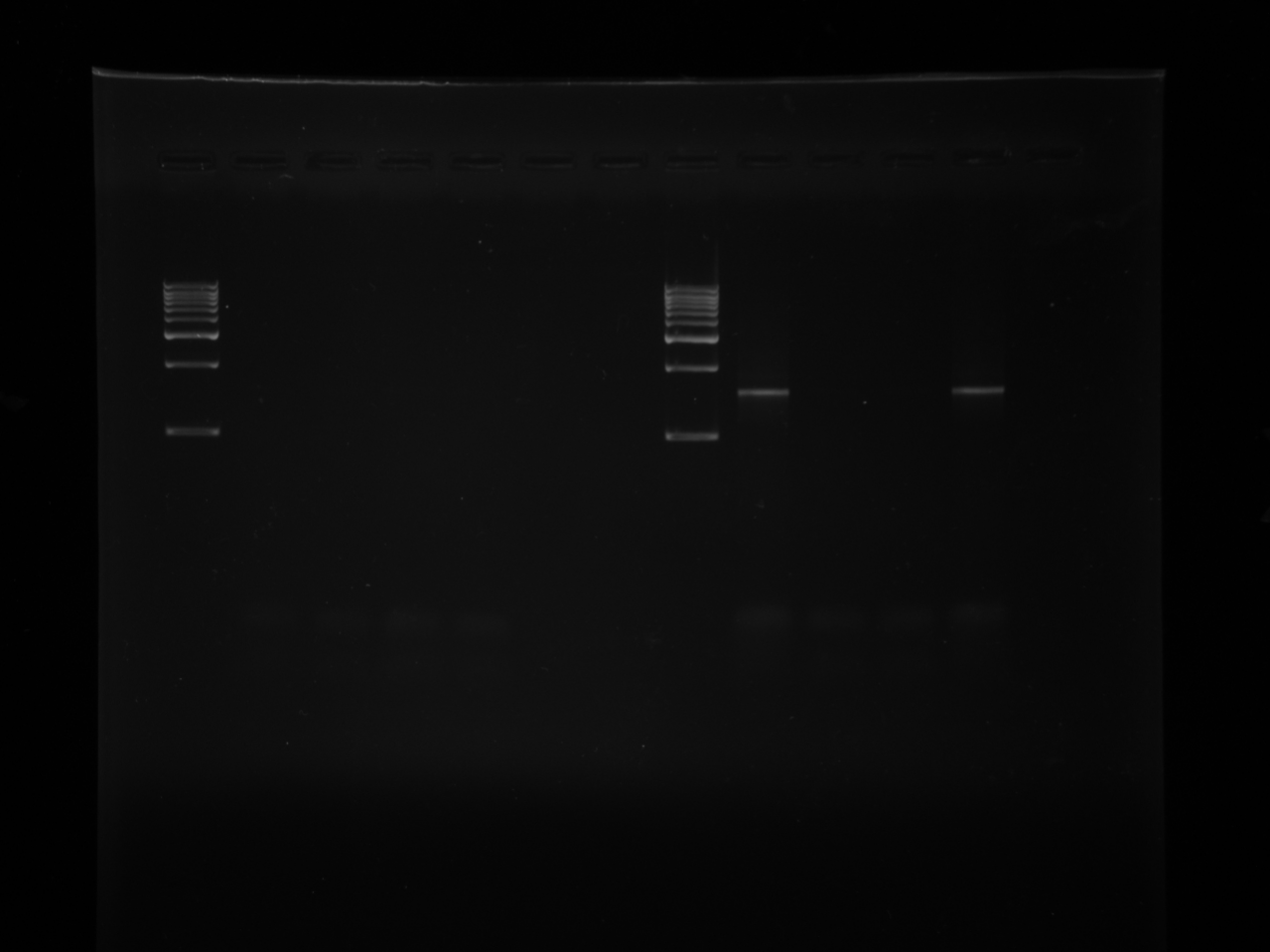

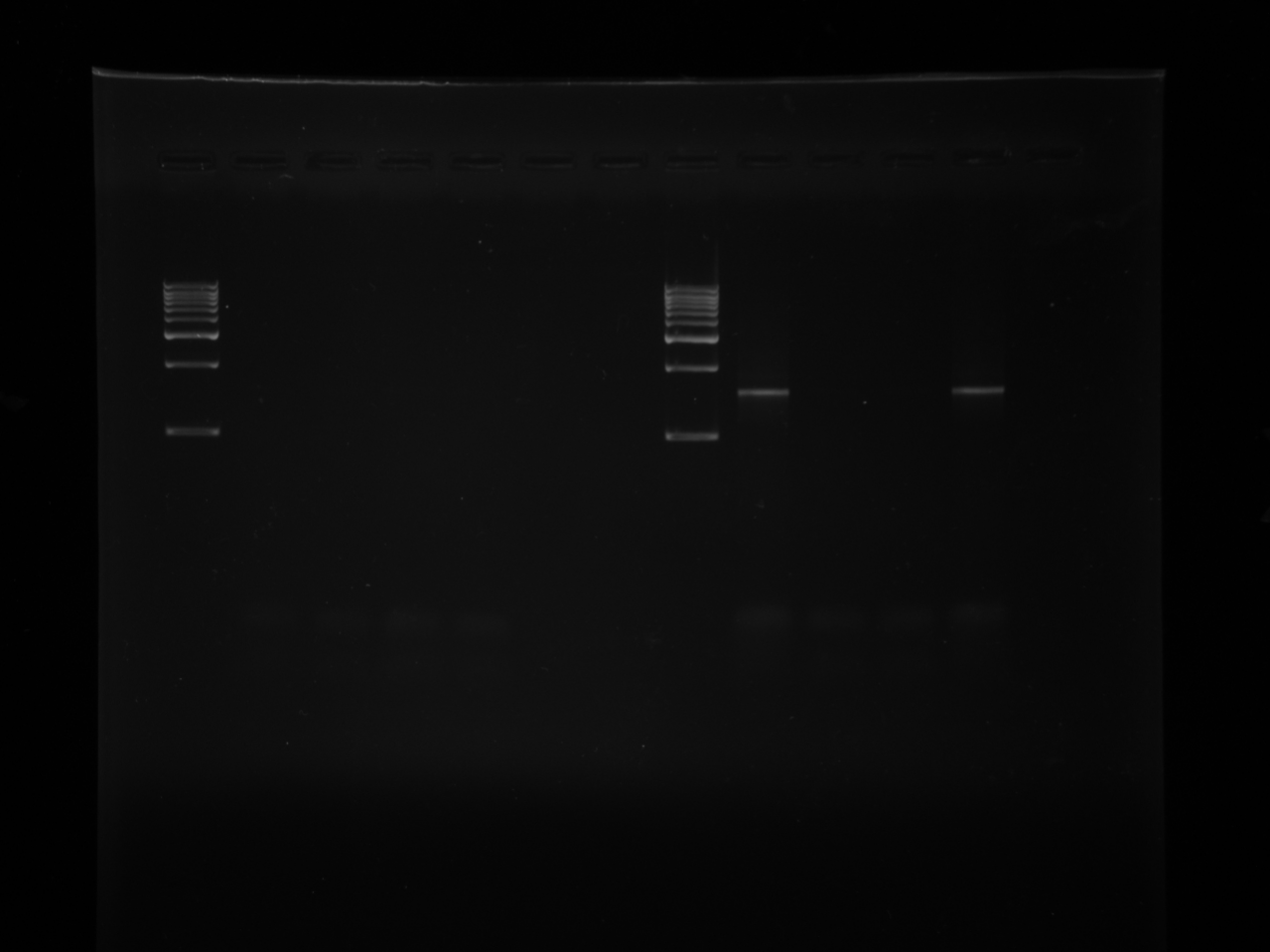


**M 1 2 3 4 5**

**M 1 2 3 4 5**

**M 1 2 3 4 5**

**M 1 2 3 4 5**

**M 1 2 3 4 5**

**A**

**B**

**C**

**D**

**E**

**S3 Fig. Gel electrophoresis of PCR amplified 16S rDNA for the DNA extracted by methods M1 to M5 A.** M1**; B.** M2**; C.** M3**; D:** M4**; E:** M5**.** Samples were analyzed on 1 % agarose gel in 0.5X TBE buffer. Lane M: 1Kb DNA ladder (Merck, India); Lane 1: Garden soil; Lane 2: Sewage sludge; Lane 3: Lake soil; Lane 4: Compost. Lane 5: Negative control.
