# Supplementary material for: A Rapid and Economical Method for Efficient DNA Extraction from Diverse Soils Suitable for Metagenomic Applications
Source: PLoS One. 2015 Jul 13;10(7):e0132441. doi: 10.1371/journal.pone.0132441 (PMC4500551; doi:10.1371/journal.pone.0132441)
Supplement: S4 Fig — (DOC) [file pone.0132441.s004.doc]

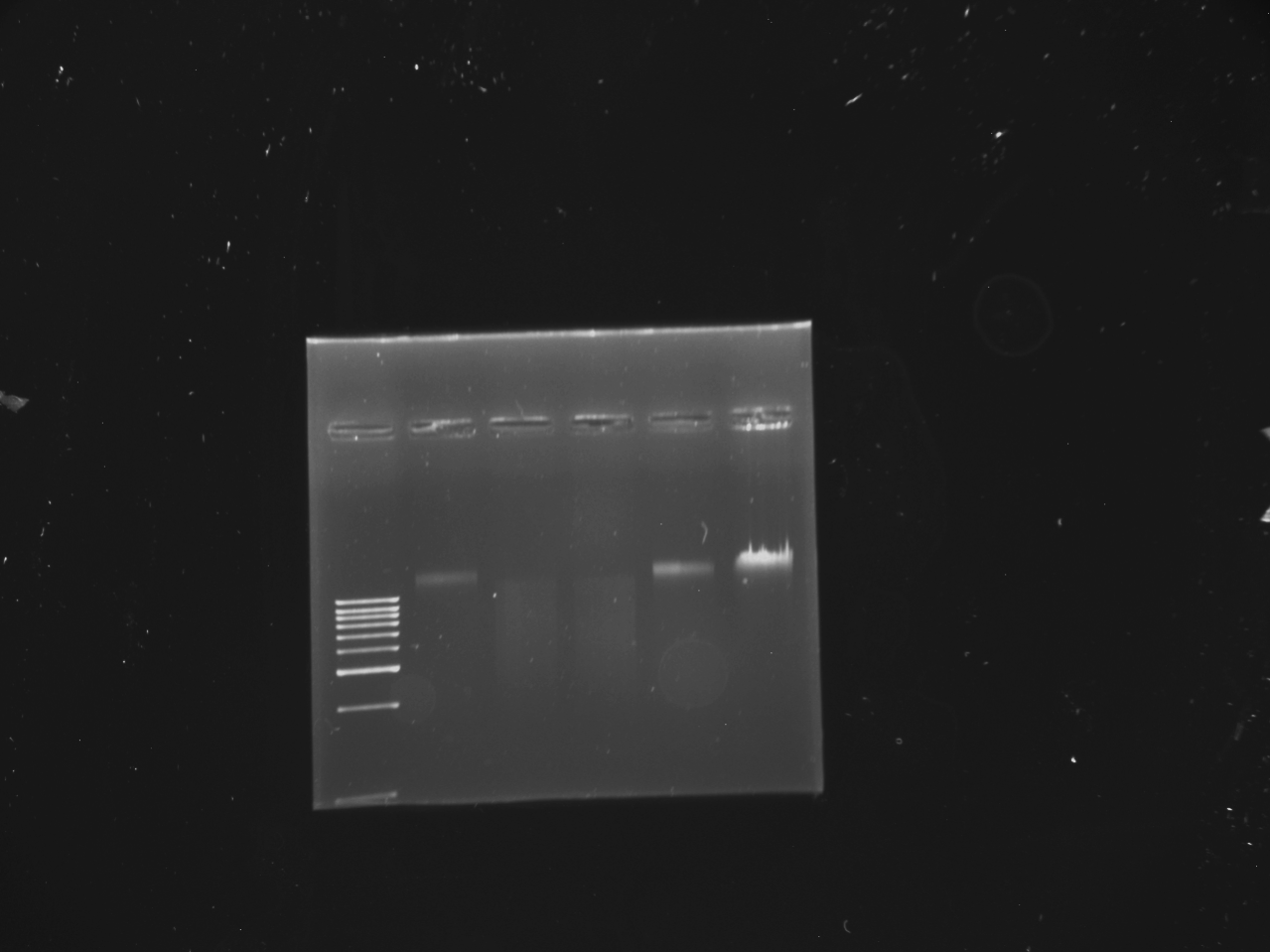

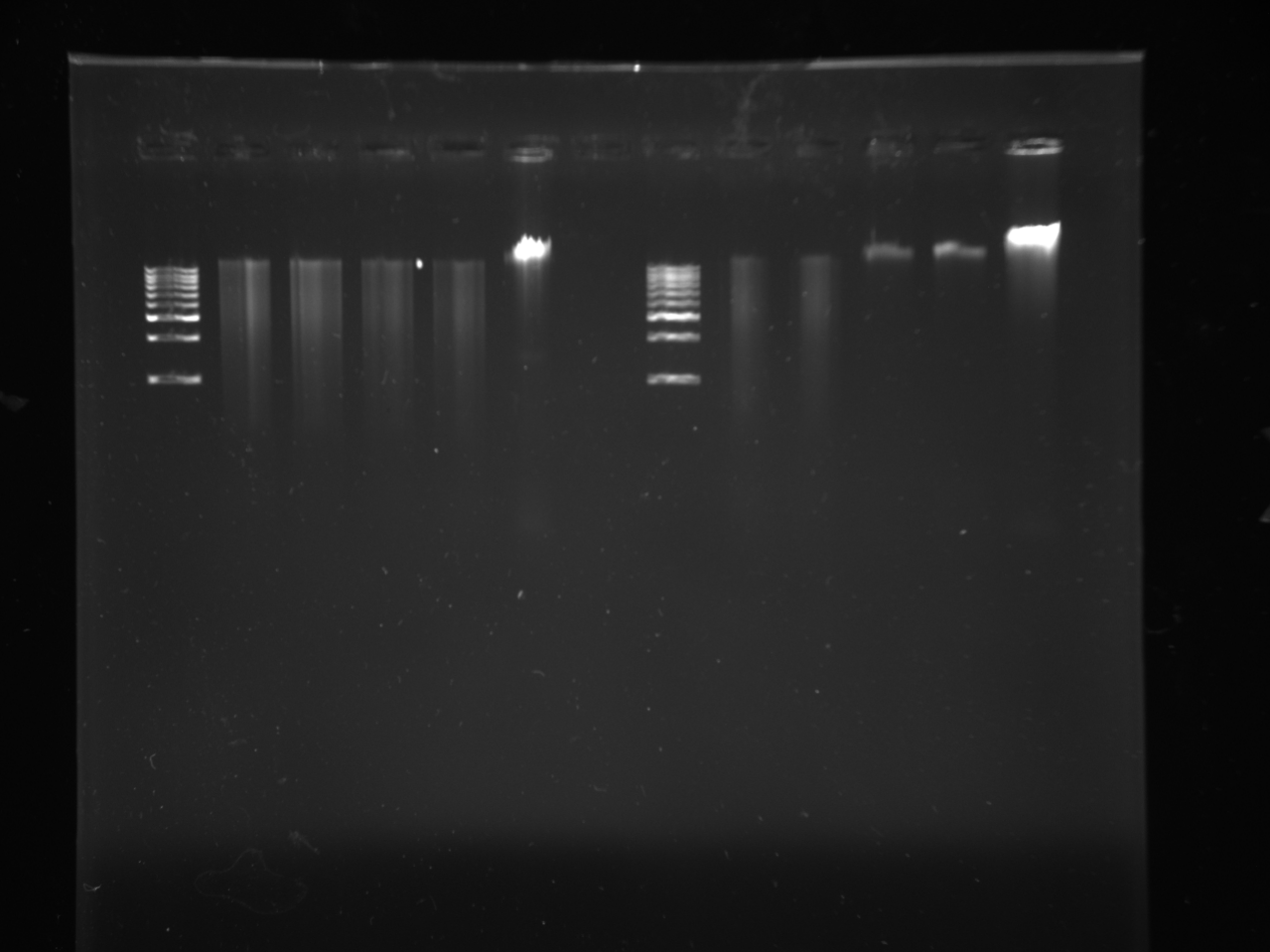

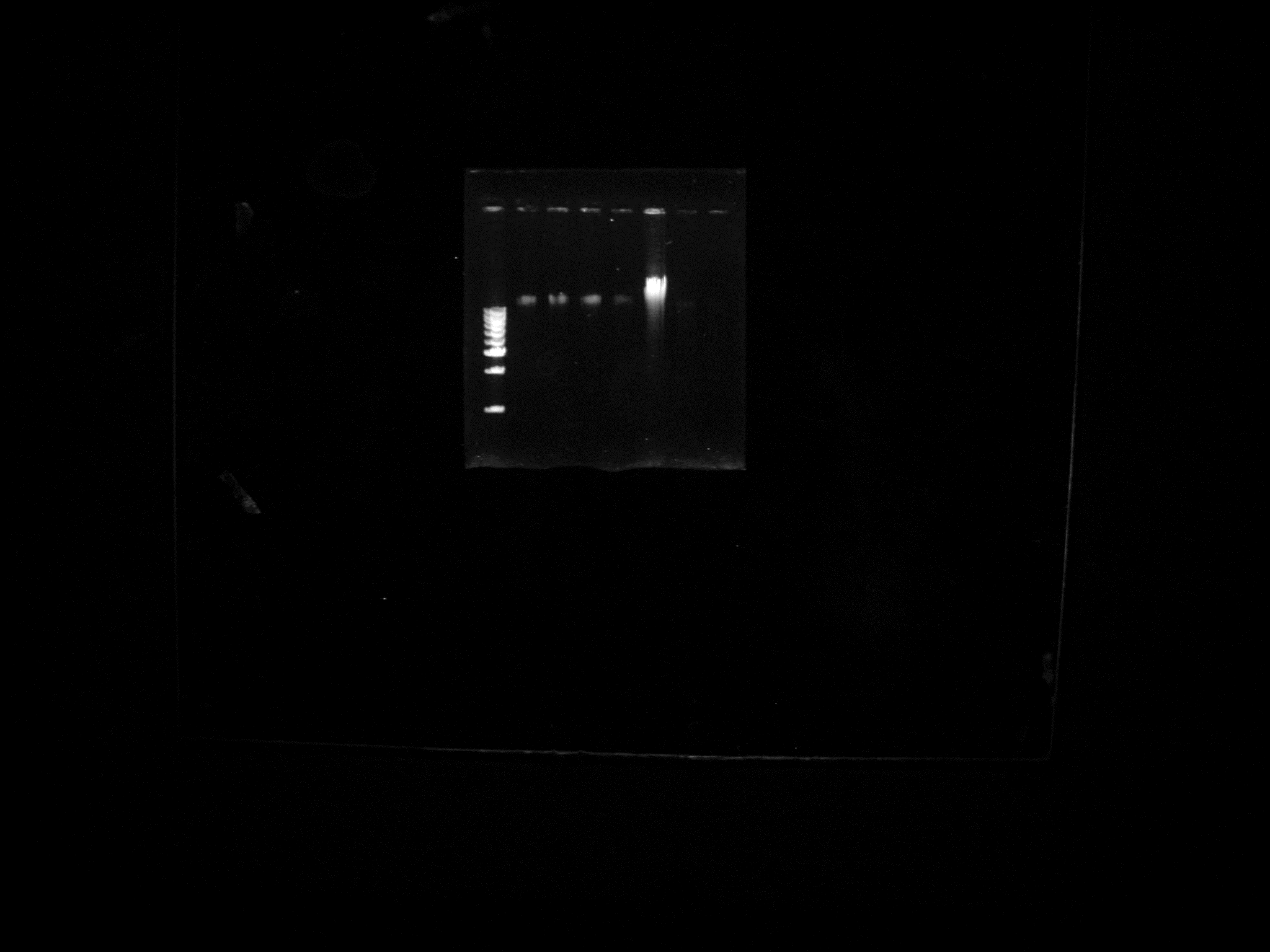

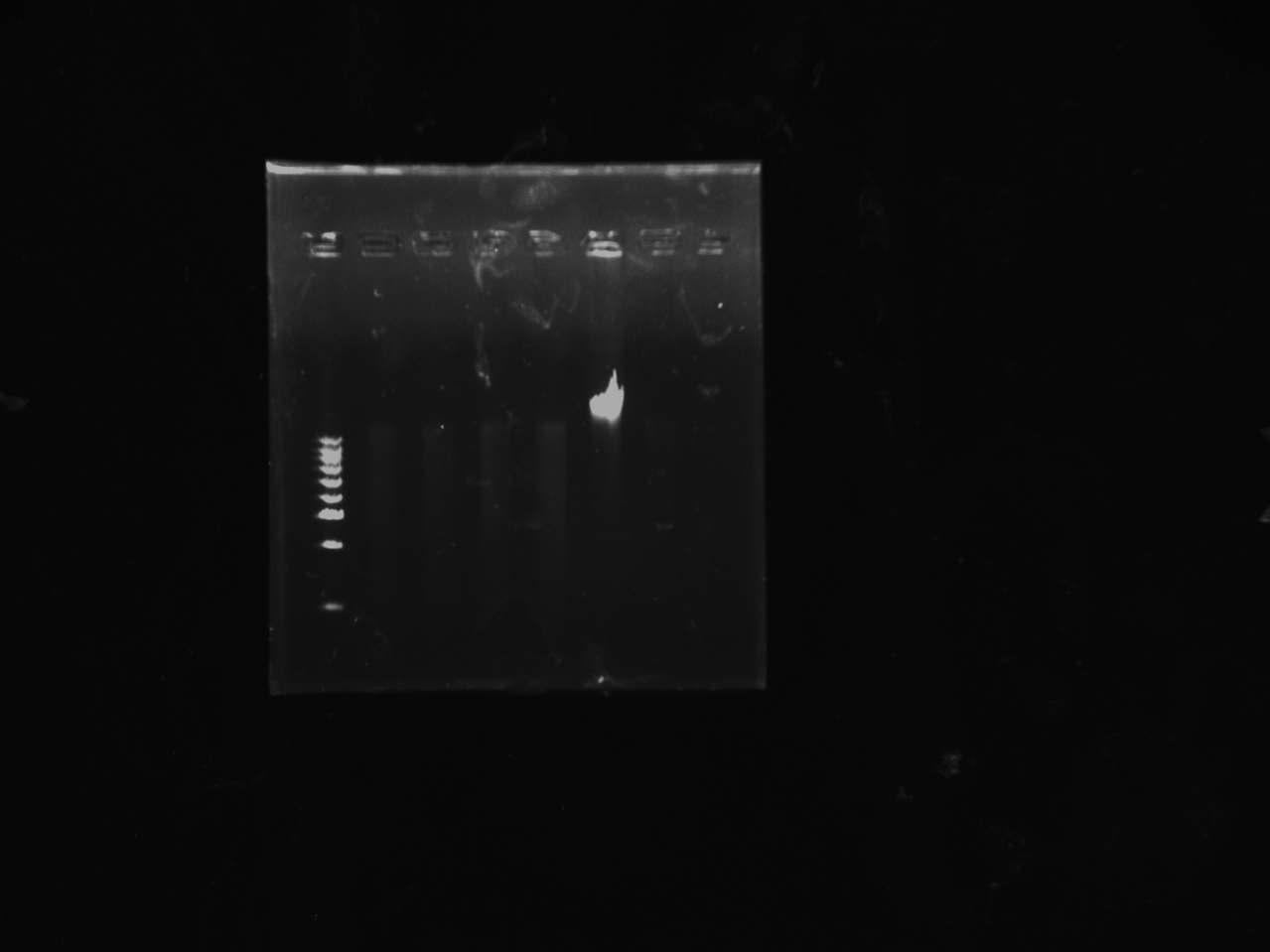


**M 1 2 3 4 5**

**M 1 2 3 4 5**

**M 1 2 3 4 5**

**M 1 2 3 4 5**

**A**

**B**

**C**

**D**

**S4 Fig. Gel electrophoresis of partial restriction digestion of the metagenomic DNA extracted by methods M1, M2, M4 and M5 using *Bam*HI. A.** M1; **B.** M2; **C.** M4; **D:** M5**.** Samples were analyzed on 0.8% agarose gel in 0.5X TBE buffer. Lane M: 1Kb DNA ladder (Merck, India); Lane 1: Garden soil; Lane 2: Sewage sludge; Lane 3: Lake soil; Lane 4: Compost. Lane 5: Undigested DNA. Method M3 was not subjected to restriction digestion because of the sheared DNA.
