# Supplementary material for: A Rapid and Economical Method for Efficient DNA Extraction from Diverse Soils Suitable for Metagenomic Applications
Source: PLoS One. 2015 Jul 13;10(7):e0132441. doi: 10.1371/journal.pone.0132441 (PMC4500551; doi:10.1371/journal.pone.0132441)
Supplement: S5 Fig — (DOC) [file pone.0132441.s005.doc]

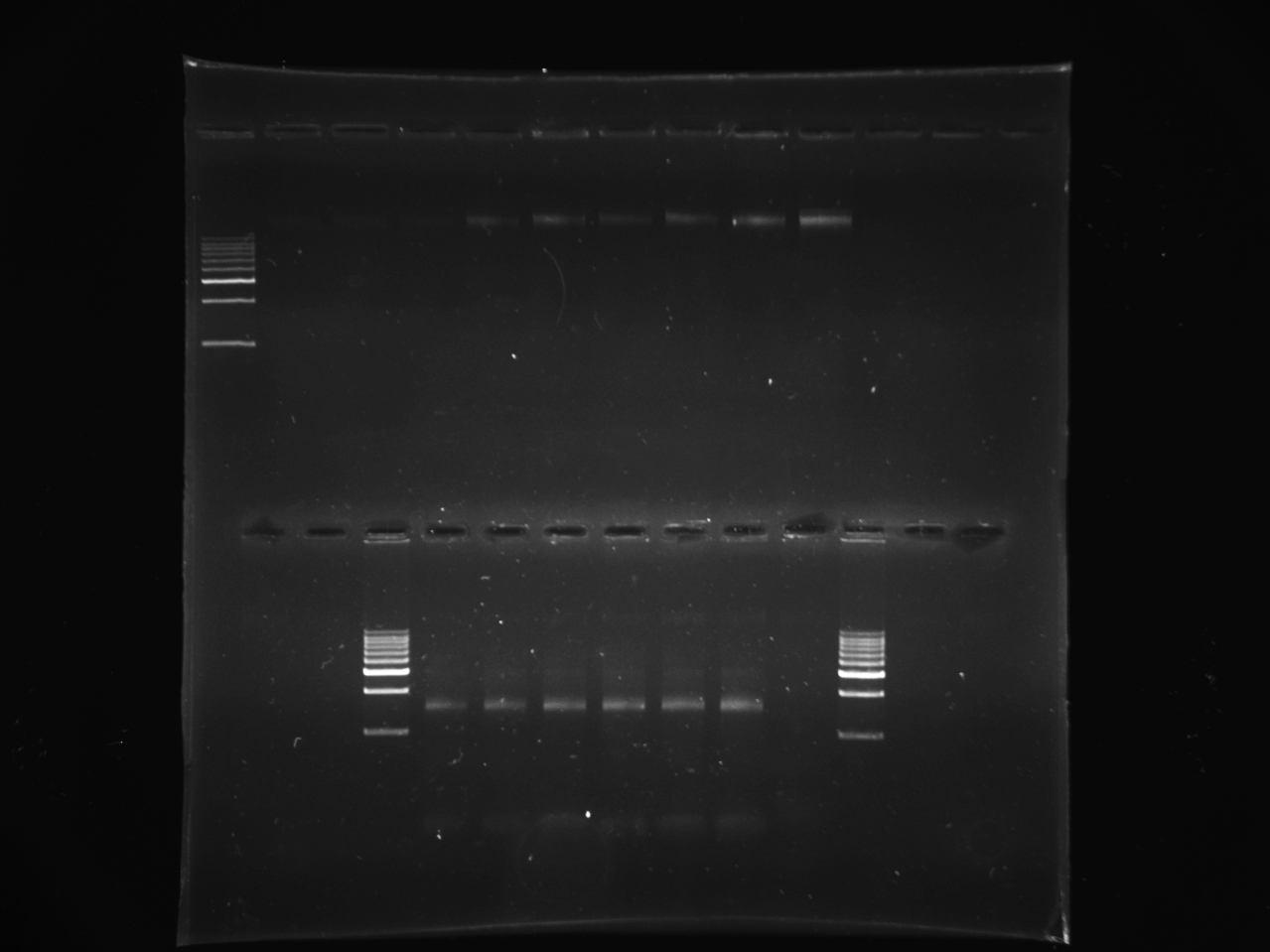

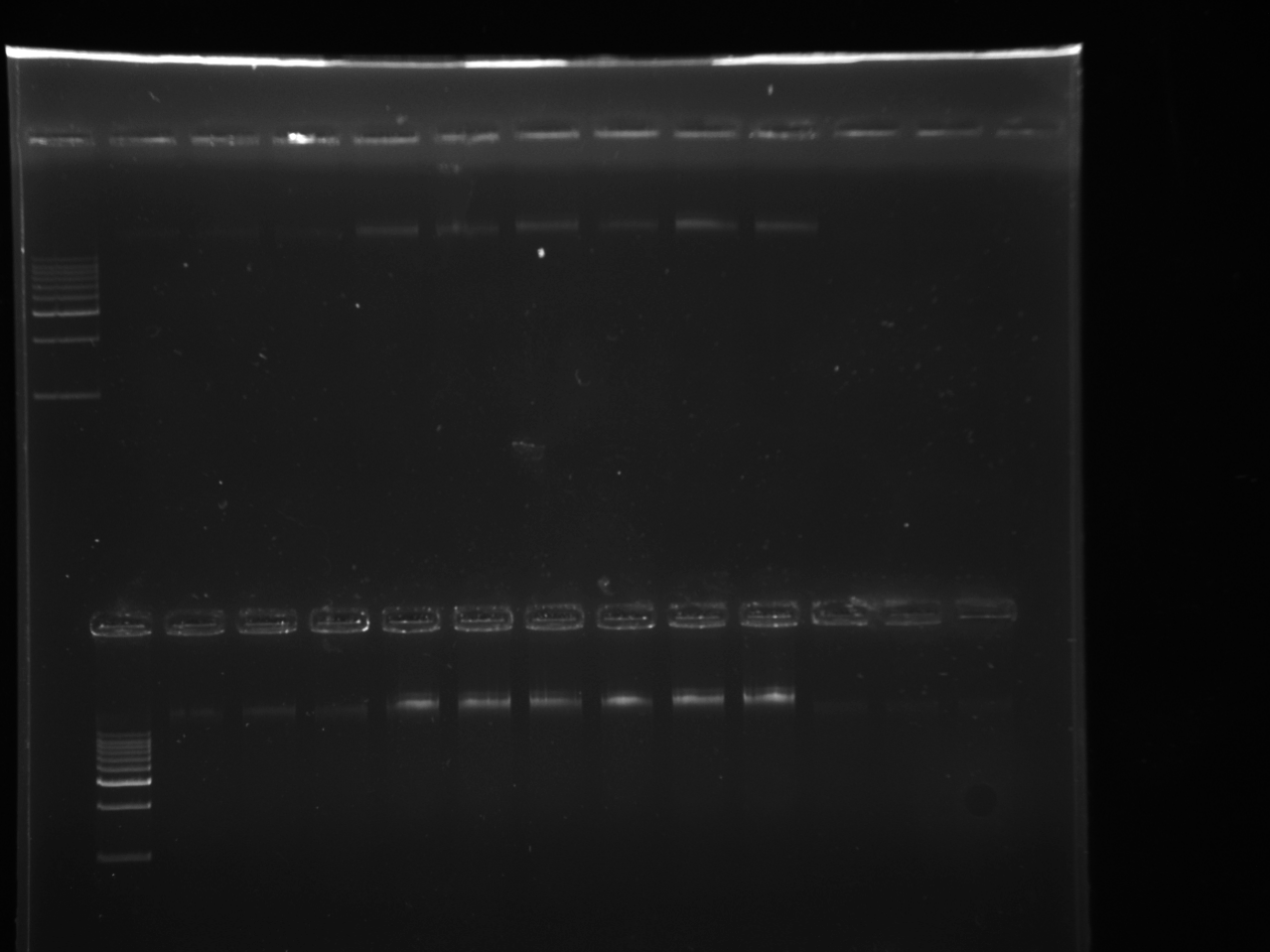

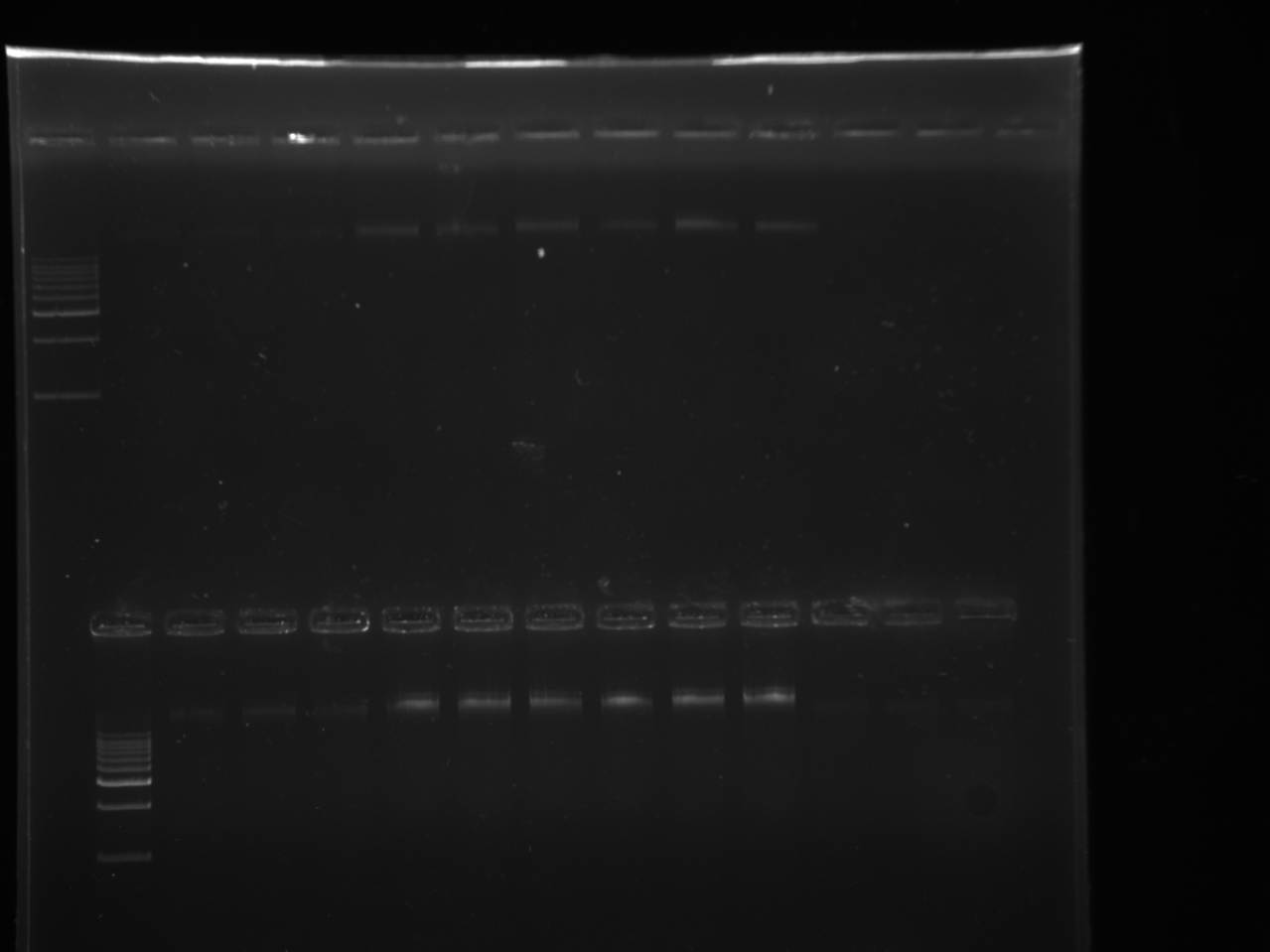

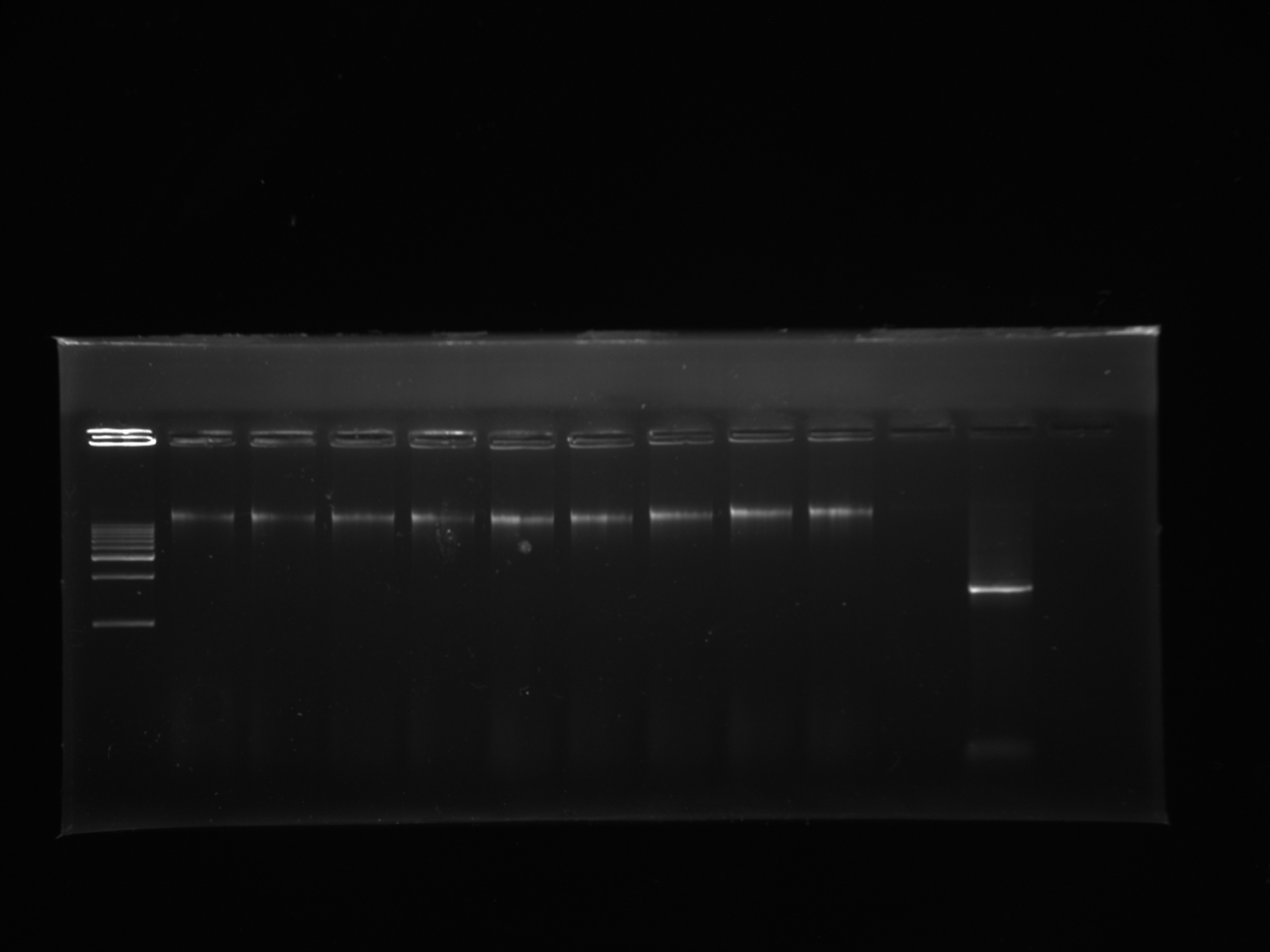


**A**

**B**

**C**

**D**

**M 1a 1b 1c 2a 2b 2c 3a 3b 3c**

**S5 Fig. Gel electrophoresis of metagenomic DNA extraction by the glass beads, glass powder and glass powder + powdered activated charcoal for four soils.** Samples were electrophoresed on 0.8% agarose gel in 0.5X TBE buffer. **A.** Garden Soil; **B**. Sewage sludge; **C.** Lake soil; **D.** Compost; Lane M represents 1Kb DNA ladder (Merck, India). Lane 1: glass beads; Lane 2: glass powder; Lane 3: glass powder + powdered activated charcoal. a,b,c represents the triplicates of corresponding method.
