# Supplementary material for: A Rapid and Economical Method for Efficient DNA Extraction from Diverse Soils Suitable for Metagenomic Applications
Source: PLoS One. 2015 Jul 13;10(7):e0132441. doi: 10.1371/journal.pone.0132441 (PMC4500551; doi:10.1371/journal.pone.0132441)
Supplement: S6 Fig — (DOC) [file pone.0132441.s006.doc]

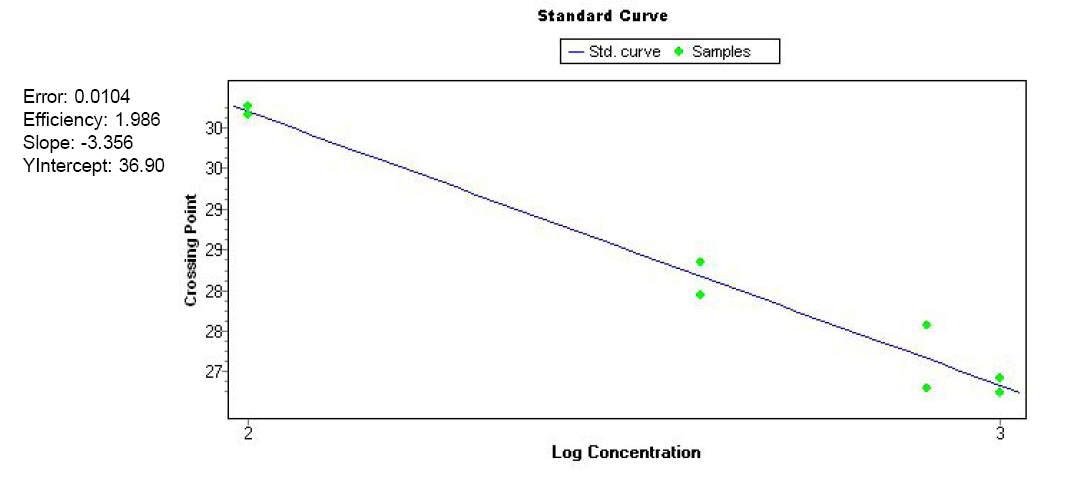

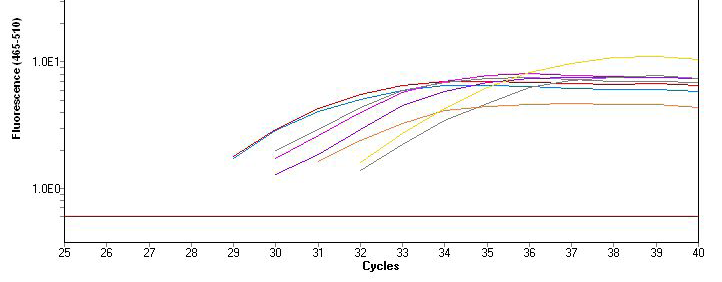


**A**

**B**

**S6 Fig. qPCR analysis for method M6. A**: The qPCR reaction graph with fluorescence against cycles. **B**: PCR efficiency standard graph for the template prepared using method M6. The PCR efficiency is 1.986 against 2 which is equal to 99.3 percent.
