# Supplementary material for: A Rapid and Economical Method for Efficient DNA Extraction from Diverse Soils Suitable for Metagenomic Applications
Source: PLoS One. 2015 Jul 13;10(7):e0132441. doi: 10.1371/journal.pone.0132441 (PMC4500551; doi:10.1371/journal.pone.0132441)
Supplement: S1 Table — (DOC) [file pone.0132441.s007.doc]

**S1 Table.**  **Characteristics of the four soil samples**

| **Sample** | **Texture** | **pH** | **EC**  **(d sm-1)** | **Organic matter**  **(%)** | **Carbon**  **(%)** | **Iron**  **(%)** | **Cadmium**  **mg/100g** | **Chromium**  **mg/100g** |
| --- | --- | --- | --- | --- | --- | --- | --- | --- |
| Garden soil | Sandy loam | 7.14 | 0.261 | 56.82 | 70.2 | 35.38 | 36.85 | 37.80 |
| Sewage  sludge | Sandy loam | 8.10 | 0.35 | 62.89 | 89.1 | 52.40 | 61.82 | 56.46 |
| Lake soil | Clay loam | 8.64 | 0.36 | 72.56 | 74.25 | 51.32 | 38.69 | 42.71 |
| Compost | Sandy loam | 7.00 | 0.21 | 68.96 | 72.23 | 45.63 | 48.56 | 45.87 |
