# Supplementary material for: A Rapid and Economical Method for Efficient DNA Extraction from Diverse Soils Suitable for Metagenomic Applications
Source: PLoS One. 2015 Jul 13;10(7):e0132441. doi: 10.1371/journal.pone.0132441 (PMC4500551; doi:10.1371/journal.pone.0132441)
Supplement: S2 Table — (DOC) [file pone.0132441.s008.doc]

**S2 Table.** Suitability of the DNA samples extracted by methods M1 to M6 for 16S rDNA PCR amplication and partial restriction digestion by *Bam*H1

| **Methods** | 1**6S rDNA PCR amplification** | **Partial restriction digestion by *Bam*H1** |
| --- | --- | --- |
|  | Garden Sewage Lake Compost  Soil sludge soil | Garden Sewage Lake Compost  Soil sludge soil |
| M1 | - - - - | - - - - |
| M2 | + - - + | + + - - |
| M3 | - - - - | - - - - |
| M4 | - - - - | - + + - |
| M5 | + - - - | + + + + |
| M6 | + + + + | + + + + |

+ indicates the methods suitable for PCR and endonuclease activity.

- indicates the methods not suitable for PCR and endonuclease activity.
