# Supplementary material for: A Rapid and Economical Method for Efficient DNA Extraction from Diverse Soils Suitable for Metagenomic Applications
Source: PLoS One. 2015 Jul 13;10(7):e0132441. doi: 10.1371/journal.pone.0132441 (PMC4500551; doi:10.1371/journal.pone.0132441)
Supplement: S3 Table — (DOC) [file pone.0132441.s009.doc]

| **Method** | **Garden soil** | **Sewage sludge** | **Lake soil** | **Compost** |
| --- | --- | --- | --- | --- |
| **M1** | 1.32, 1.35, 1.33 | 1.47, 1.52, 1.48 | 1.42, 1.44, 1.39 | 0.92, 0.96, 0.92 |
| **M2** | 4.89, 4.9, 4.95 | 4.97, 5.02, 5.06 | 1.5, 1.58, 1.51 | 2.31, 2.21, 2.20 |
| **M3** | 3.69, 3.57, 3.63 | 0.51, 0.54, 0.62 | 1.06, 1.10, 1.07 | 0.59, 0.62, 0.66 |
| **M4** | 5.01, 5.12, 5.06 | 2.0, 2.13, 2.05 | 2.29, 2.21, 2.26 | 4.21, 4.13, 4.10 |
| **M5** | 1.98, 2.04, 2.01 | 2.57, 2.52, 2.61 | 4.57, 4.56, 4.61 | 4.92, 4.89, 4.86 |
| **M6** | 5.52, 5.47, 5.45 | 7.82, 7.85, 7.76 | 5.25, 5.12, 5.19 | 5.03, 4.94, 4.98 |

**S3 Table. Triplicate values for DNA DNA yield (µg/g of soil) for methods M1 to M6.**
