# Supplementary material for: A Rapid and Economical Method for Efficient DNA Extraction from Diverse Soils Suitable for Metagenomic Applications
Source: PLoS One. 2015 Jul 13;10(7):e0132441. doi: 10.1371/journal.pone.0132441 (PMC4500551; doi:10.1371/journal.pone.0132441)
Supplement: S4 Table — (DOC) [file pone.0132441.s010.doc]

| **Method** | **Garden soil** | **Sewage sludge** | **Lake soil** | **Compost** |
| --- | --- | --- | --- | --- |
| **M1** | 1.12, 1.15, 1.16 | 1.19, 1.24, 1.23 | 1.30, 1.13, 1.23 | 1.25, 1.32, 1.30 |
| **M2** | 1.50, 1.51, 1.60 | 1.51, 1.45, 1.49 | 1.55, 1.55, 1.57 | 1.33, 1.47, 1.40 |
| **M3** | 1.35, 1.33, 1.29 | 1.19, 1.30, 1.27 | 1.35, 1.33, 1.34 | 1.30, 1.38, 1.42 |
| **M4** | 1.29, 1.38, 1.32 | 1.32, 1.29, 1.40 | 1.49, 1.36, 1.53 | 1.48, 1.44, 1.51 |
| **M5** | 1.62, 1.57, 1.65 | 1.70, 1.54, 1.62 | 1.52, 1.56, 1.59 | 1.43, 1.57, 1.52 |
| **M6** | 1.75, 1.80, 1.91 | 1.69, 1.77, 1.71 | 1.80, 1.77, 1.76 | 1.72, 1.78, 1.68 |

**S4 Table. Triplicate values for A_260/280_**
