# Supplementary material for: A Rapid and Economical Method for Efficient DNA Extraction from Diverse Soils Suitable for Metagenomic Applications
Source: PLoS One. 2015 Jul 13;10(7):e0132441. doi: 10.1371/journal.pone.0132441 (PMC4500551; doi:10.1371/journal.pone.0132441)
Supplement: S5 Table — (DOC) [file pone.0132441.s011.doc]

| **Method** | **Garden soil** | **Sewage sludge** | **Lake soil** | **Compost** |
| --- | --- | --- | --- | --- |
| **M1** | 0.09,0.07, 0.12 | 0.06, 0.08, 0.09 | 0.09, 0.06, 0.12 | 0.09, 0.08, 0.07 |
| **M2** | 0.07,0.08, 0.03 | 0.04, 0.07, 0.09 | 0.09, 0.06, 0.08 | 0.05, 0.02, 0.10 |
| **M3** | 0.06, 0.07, 0.09 | 0.09, 0.10, 0.05 | 0.02, 0.05, 0.10 | 0.02, 0.05, 0.12 |
| **M4** | 0.06, 0.09, 0.13 | 0.06, 0.08, 0.09 | 0.05, 0.09, 0.11 | 0.11, 0.07, 0.08 |
| **M5** | 0.02, 0.05, 0.10 | 0.07, 0.09, 0.04 | 0.01, 0.11, 0.13 | 0.07, 0.09, 0.08 |
| **M6** | 0.04, 0.06, 0.01 | 0.05, 0.02, 0.10 | 0.06, 0.04, 0.06 | 0.01, 0.01, 0.12 |

**S5 Table. Triplicate values for A_340_**
