# Supplementary material for: A Rapid and Economical Method for Efficient DNA Extraction from Diverse Soils Suitable for Metagenomic Applications
Source: PLoS One. 2015 Jul 13;10(7):e0132441. doi: 10.1371/journal.pone.0132441 (PMC4500551; doi:10.1371/journal.pone.0132441)
Supplement: S6 Table — (DOC) [file pone.0132441.s012.doc]

| **Method** | **Garden soil** | **Sewage sludge** | **Lake soil** | **Compost** |
| --- | --- | --- | --- | --- |
| **Glass beads** | 3.27, 3.36, 3.29 | 1.09, 1.11, 1.14 | 0.97, 0.89, 1.02 | 2.92, 2.99, 3.26 |
| **Glass powder** | 5.44, 5.49, 5.57 | 5.76, 5.82, 5.9 | 3.98, 4.26, 4.23 | 3.97, 4.08, 4.27 |
| **Glass powder + PAC** | 6.18, 6.2, 6.26 | 5.81, 5.87, 5.87 | 4.43, 4.36, 4.38 | 4.88, 4.97, 5.06 |

**S6 Table. Triplicate values for DNA yield (µg/g of soil) for method M2 and its modification**
